# Improved object detection method for unmanned driving based on Transformers

**DOI:** 10.3389/fnbot.2024.1342126

**Published:** 2024-05-01

**Authors:** Huaqi Zhao, Xiang Peng, Su Wang, Jun-Bao Li, Jeng-Shyang Pan, Xiaoguang Su, Xiaomin Liu

**Affiliations:** ^1^The Heilongjiang Provincial Key Laboratory of Autonomous Intelligence and Information Processing, School of Information and Electronic Technology, Jiamusi University, Jiamusi, China; ^2^Harbin Institute of Technology, Harbin, China; ^3^School of Artificial Intelligence, Nanjing University of Information Science and Technology, Nanjing, China

**Keywords:** object detection, feature extraction, query denoising, optimal transport, Transformer

## Abstract

The object detection method serves as the core technology within the unmanned driving perception module, extensively employed for detecting vehicles, pedestrians, traffic signs, and various objects. However, existing object detection methods still encounter three challenges in intricate unmanned driving scenarios: unsatisfactory performance in multi-scale object detection, inadequate accuracy in detecting small objects, and occurrences of false positives and missed detections in densely occluded environments. Therefore, this study proposes an improved object detection method for unmanned driving, leveraging Transformer architecture to address these challenges. First, a multi-scale Transformer feature extraction method integrated with channel attention is used to enhance the network's capability in extracting features across different scales. Second, a training method incorporating Query Denoising with Gaussian decay was employed to enhance the network's proficiency in learning representations of small objects. Third, a hybrid matching method combining Optimal Transport and Hungarian algorithms was used to facilitate the matching process between predicted and actual values, thereby enriching the network with more informative positive sample features. Experimental evaluations conducted on datasets including KITTI demonstrate that the proposed method achieves 3% higher mean Average Precision (mAP) than that of the existing methodologies.

## 1 Introduction

Unmanned driving, a process where vehicles employ sensors to perceive their surroundings, make real-time driving decisions, avoid obstacles, and complete driving tasks without human intervention, relies heavily on accurate object detection for safe operation (Li G. et al., 2022). Despite technological advancements, challenges persist in the practical application of object detection methods in unmanned driving technology.

Object detection methods encompass both traditional and deep learning-based object detection approaches. Traditional methods adopt sliding windows to obtain candidate boxes, followed by feature extraction using techniques such as Histogram of Oriented Gradient (HOG) (Dalal and Triggs, 2005) and classification using algorithms such as Support Vector Machine (SVM) (Cortes and Vapnik, 1995). However, these methods suffer from poor detection performance and high computational complexity owing to manual feature region selection. In the era of deep learning, Convolutional Neural Network (CNN)-based object detection methods are widely used. They are classified into one- and two-stage algorithms based on their detection strategies, differing in the generation of candidate regions. In 2014, Girshick et al. proposed the Region-Convolutional Neural Network (R-CNN), a significant advancement over traditional methods, demonstrating improved accuracy and speed (Girshick et al., 2014). Subsequent developments, such as Dynamic R-CNN, further enhanced accuracy (Zhang et al., 2020); however, speed remained a concern. Therefore, one-stage object detection algorithms have been proposed to address speed limitations. For example, the Yolo Only Look Once (YOLO) algorithm, proposed in 2016, directly outputs detection results without generating candidate regions, significantly improving detection speed (Redmon et al., 2016). This approach has become a hallmark of one-stage detection algorithms. Various iterations, including the Single Shot MultiBox Detector (SSD) (Liu et al., 2016) and subsequent versions of YOLO series, have since been developed and continually refined (Yung et al., 2022) YOLOv7, for instance, combines various methods to enhance both accuracy and speed, finding wide application in unmanned driving and real-time monitoring (Wang et al., 2023).

However, object detection methods based on CNNs are limited by the receptive field, which makes it difficult to model the image globally. With the improvement in computing power, the data demand for this method is increasingly saturated. The Transformer-based object detection methods rely on powerful global modeling and data fitting abilities, which are gradually emerging in the domain of object detection. Carion et al. proposed the Detection Transformer (DETR) object detection algorithm in 2020, which laid the foundation for the application of the Transformer in the domain of object detection. DETR regards the object detection task as a set prediction problem and uses the Hungarian algorithm to match predicted and ground truth objects, avoiding post-processing operations such as Non-Maximum Suppression (NMS) (Carion et al., 2020). Zhu et al. proposed Deformable-DETR in 2021 to address the high computational complexity of DETR (Zhu et al., 2020). In 2022, Wang Y. et al. (2022) introduced the Anchor-DETR algorithm, defining the query vector as the center point coordinate and inputting it into the decoder for training, thereby significantly improving model convergence speed. Building upon Anchor-DETR, Dynamic Anchor Box-DETR (DAB-DETR) incorporates query vector adjustments including the center point coordinate, height, and width of the anchor box, alongside an Anchor-update strategy to further improve detection accuracy (Liu S. et al., 2022). DeNoising-DETR (DN-DETR) addresses Hungarian matching instability, which impedes model convergence, by proposing a query vector denoising method that significantly accelerates convergence speed (Li F. et al., 2022). DETR with improved deNoising anchOr boxes (DINO) integrates the advantages of preceding algorithms and introduces a comparative denoising method, demonstrating promising results on the COCO dataset (Zhang et al., 2022). Additionally, Li F. et al. (2023) present Lite DETR, a simple yet efficient end-to-end object detection framework capable of reducing the GFLOPs of the detection head by 60% while maintaining 99% of the original performance.

In natural images, the aforementioned object detection method performs satisfactorily. However, when applied to unmanned driving images, the method encounters the following challenges:

1. Feature extraction methods employing CNNs are limited by the receptive field. Therefore, extracting feature information from multi-scale changing objects becomes challenging.
2. Owing to the low resolution of unmanned driving images, the available feature information within the images is insufficient. Additionally, pixels representing small objects are rare, resulting in a decrease in the accuracy of small object detection.
3. The mutual occlusion of vehicles, pedestrians, and other objects object poses a challenge in distinguishing features. Consequently, this leads to missed and false detections, particularly in densely occluded scenarios.

Addressing the challenges in object detection for unmanned driving, we propose an improved method based on Transformers, incorporating three key improvements:

1. In response to suboptimal performance for multi-scale changing objects, we investigate a multi-scale Transformer feature extraction method fused with channel attention. This method uses the Transformer model to obtain the feature information of multi-scale change objects, while the channel attention module weights channel features to improve detection performance in such scenarios.
2. To address the problem of low accuracy in detecting small objects, we employ a training method for query denoising with Gaussian decay to train the network. This method uses a Gaussian decay function to construct a multi-scale ground truth (GT) box with different noise levels, which is subsequently converted into a multi-scale query vector input model for denoising training. By introducing significant noise to small objects, the model learns more positive and negative sample features of small objects during the noise reduction process, thereby promoting the detection accuracy of small objects.
3. To resolve the issue of missed and false detections of densely occluded objects, we implement a hybrid matching method based on optimal transport and Hungarian algorithms to conduct the matching process between predicted and ground truth objects. In the training phase, this hybrid matching method is used to expand the number of positive samples, enabling the model to obtain additional feature information from positive samples. This augmentation helps in resolving the issue of missed and false detections in dense occlusion scenarios.

## 2 Related work

### 2.1 Transformer-based feature extraction method

In 2020, Google introduced the Vision Transformer (ViT) image classification model, which was the first application of the Transformer for image classification (Dosovitskiy et al., 2020). Compared with a CNN, the Transformer has a larger receptive field and global modeling capability of features. Therefore, the feature extraction network based on the Transformer can better extract image features. In 2020, Beal et al. proposed a Version Transformer-Faster Convolutional Neural Network (ViT-FRCNN) object detection algorithm. The algorithm uses a Transformer-based feature extraction network to replace the CNN-based feature extraction network and achieves optimal detection results (Beal et al., 2020). However, when capturing high-resolution images, the processing speed of the Transformer is not ideal owing to its high computational complexity. Therefore, Liu et al. proposed Shifted Windows Transformer (Swin-Transformer) in 2021, which introduces a local attention mechanism. The attention computation is restricted within the window, significantly reducing the computational cost (Liu et al., 2021). Wang et al. (2021) proposed Pyramid Vision Transformer (PVT) from the perspective of input downsampling, which reduced the computational complexity by hierarchical image reduction. In the following year, Swin-Transformer _v2 (Liu Z. et al., 2022) and PVT _v2 (Wang W. et al., 2022) were proposed, further improving the feature extraction performance and decreasing computational complexity. Despite the Transformer requiring high hardware computing power, the receptive field of the Transformer is substantial. This indicates that the feature extraction ability of multiscale changing objects still needs improvement.

### 2.2 Attention mechanism

The attention mechanism focuses the model's attention on the crucial regions by weighting features to improve the performance of the image-processing task. Squeeze-and-Excitation Network (SE-Net) learns to feature weights through the loss function, enlarges the weights of important features, and improves the network expression ability (Hu et al., 2018). For the first time, Frequency Channel Attention (FCA-Net) considered the attention mechanism from the perspective of the frequency domain, combined Discrete Cosine Transform (DCT) with the channel attention mechanism, and improved the Selective Kernel Networks (SE-Net) extrusion module (Qin et al., 2021). These networks introduce the SK module, enhancing the interaction between features by evenly splitting feature maps and cross-attention. However, owing to the various hyperparameters and iterations, it requires various computing resources (Li et al., 2019). Efficient Channel Attention (ECA-Net) proposed a local cross-channel interaction module without dimensionality reduction, which has a small number of parameters and negligible computation (Wang et al., 2020). Gated Channel Transformation (GCT) improves the processing ability of large-size images by fusing global context and local information and reduces the number of parameters and calculations by adopting separable convolution (Yang et al., 2020). To better retain channel information, Ouyang et al. proposed an efficient attention module that can learn across spaces. This module reshapes part of channels in batches and categorizes them into multiple groups of sub-features for spatial semantic features to be evenly distributed in each feature group. This effectively preserves channel information and significantly reduces the amount of calculation (Ouyang et al., 2023). Addressing the challenge of the high computational complexity of the Attention module in Transformer, Zhu et al. proposed Dynamic Sparse Attention to achieve more flexible feature extraction and computation allocation. Moreover, it exhibits superior performance in small object detection tasks (Zhu et al., 2023). However, as an auxiliary method, the attention mechanism is typically combined with a convolutional network. Combination with the Transformer network is extremely crucial for the development of computer vision.

### 2.3 Optimal transport theory

Optimal transport theory describes the optimal means to transport data between two different distributions. Currently, this theory is widely adopted in the domain of image processing. Liu et al. (2020) integrated gradient weight into this algorithm as an empirical distribution to address the dense matching problem between semantically similar images. Wang S. et al. (2022) proposed a general feature selection module based on Optimal Transport to resolve the problem of model segmentation performance degradation caused by domain-independent features during unsupervised domain adaptive transfer learning. Moreover, SuperGlue employs the matching degree score of the feature matching vector to construct the Optimal Transport cost matrix and uses the Sinkhorn algorithm to solve the transport plan to complete feature point matching (Sarlin et al., 2020). In 2021, Ge et al. (2021a) proposed an Optimal Transport Assignment (OTA) algorithm to resolve the matching issue between samples and the ground truth in object detection. The algorithm utilizes Sinkhorn-Knopp to iteratively obtain an optimal transport plan. In the same year, Ge et al. (2021b) proposed the Sim-OTA matching strategy, replacing the Sinkhorn-Knopp algorithm with a simpler dynamic top-k algorithm, significantly improving the detection speed. Sun et al. (2021) proposed the Sparse Region-Convolutional Neural Network (Sparse R-CNN) object detection algorithm in 2021. The model first uses Optimal Transport matching for initial screening. Subsequently, it uses Hungarian matching to complete secondary screening, removing the NMS process. Li et al. proposed to regard inverse diffusion as the Optimal Transport problem of latent data at different stages and proposed DPM-OT. It effectively alleviates the modal mixing problem in the Diffusion Probabilistic Model, Li et al. proposed to regard inverse diffusion as the Optimal Transport problem of latent data at different stages and proposed DPM-OT. It effectively alleviates the modal mixing problem. Based on SuperGlue, Li Z. et al. (2023) decouple the similarity score from the matchability score, to effectively solve the problem of slow convergence of the Sinkhorn algorithm in training. Based on SuperGlue, Lindenberger et al. (2023) decouple the similarity score from the matchability score, to effectively address the problem of slow convergence of the Sinkhorn algorithm in training. While the optimal transport theory has become increasingly mature, numerous challenges exist when combining it with the Transformer-based object detection. Hence, this study aimed to combine it with optimal transport theory.

## 3 Improved object detection method for unmanned driving based on Transformers

**Figure 1:**
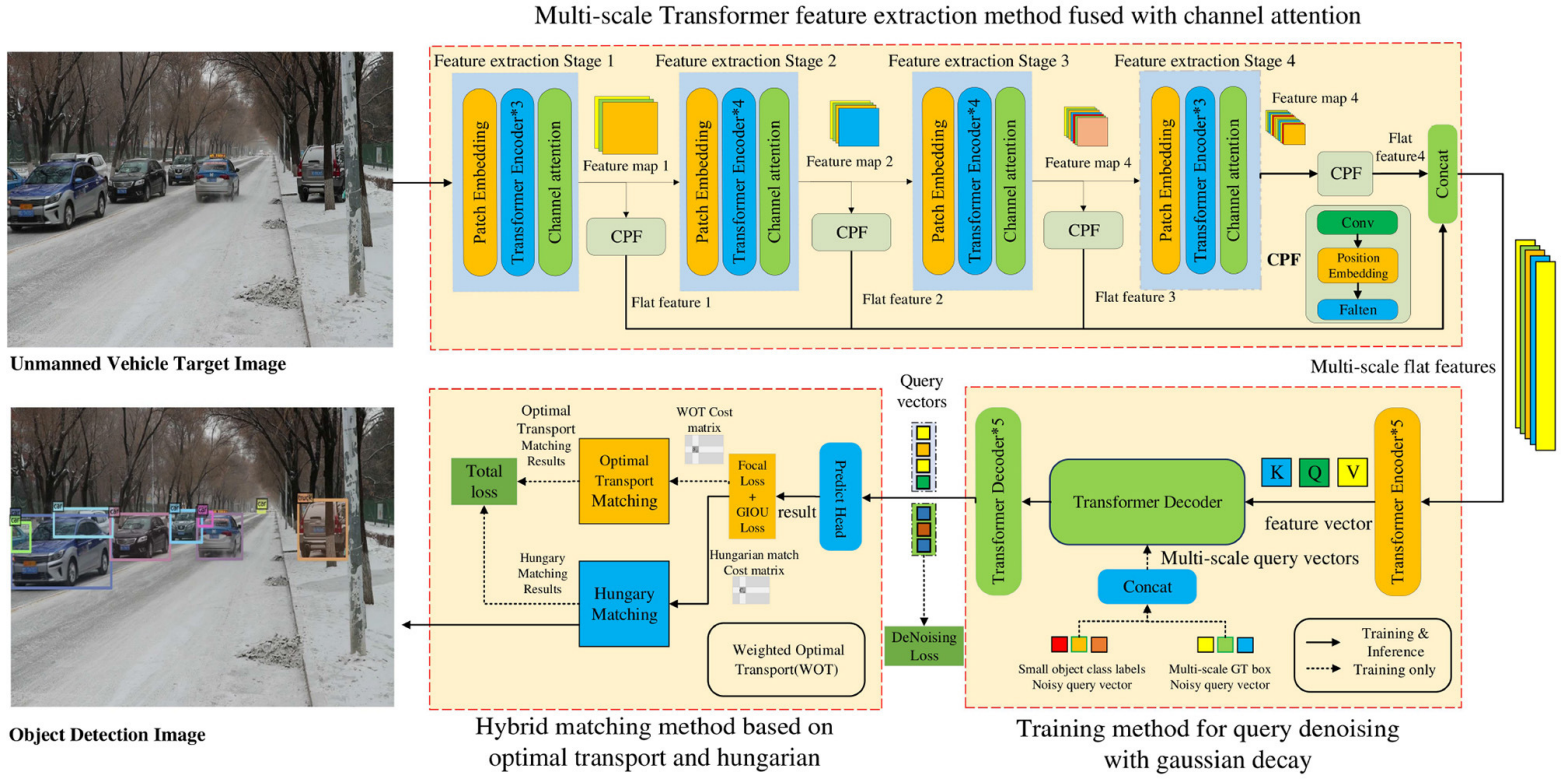
Improved object detection method for unmanned driving based on Transformers.

We propose an improved object detection method for unmanned driving utilizing Transformers, as shown in Figure 1. First, an unmanned vehicle target image is input, and the multi-scale Transformer feature extraction method fused with channel attention is adopted to extract object features. Additionally, multi-scale flat features are output to address the problem of unsatisfactory multi-scale change object detection performance. Subsequently, the multi-scale flat features are incorporated into the Transformer encoder to obtain the feature vectors (*Q, K, V*). A training method for query denoising utilizing Gaussian decay is employed to construct GT (ground truth) boxes containing different levels of noise and convert them into multi-scale query vectors. These are integrated into the Transformer decoder with the feature vector for training. The query vector is output to resolve the issue of low accuracy in small-object detection. Finally, the prediction results are obtained by decoding the query vector and a hybrid matching method based on optimal transport and Hungarian is used to output the final matching result to resolve the issue of missed and false detections of densely occluded objects. Based on the above framework, this study examined the multi-scale Transformer feature extraction method fused with channel attention, a training method for query denoising with Gaussian decay, and a hybrid matching method utilizing optimal transport and Hungarian as follows:

1. First, the multi-scale Transformer feature extraction method fused with channel attention is adopted to extract the feature information of multi-scale change objects to improve the detection performance of multi-scale change objects. This method uses the channel attention module to weight the multi-scale channel features from the Transformer network, which solves the issue of channel feature information loss in the process of multi-scale feature fusion and improves the feature extraction ability of the network for multi-scale changing objects.
2. Second, the training method for query denoising based on Gaussian decay can accelerate model convergence and boost the accuracy of small object detection. This method additionally feeds multi-scale GT bounding boxes with different noise levels into the Transformer decoder and trains the model to reconstruct the original boxes. As this method adds a large degree of noise to small objects, the model obtains more positive and negative samples, strengthens the learning capability of the model, and boosts the detection performance of these objects.
3. Third, a hybrid matching method based on optimal transport and Hungarian can help the model obtain more positive sample features. The matching results are solved by the optimal transport and Hungarian algorithms. Subsequently, the weighted loss of the two matching results is calculated to obtain the total loss, which is adopted to guide the update of network parameters.

### 3.1 Multi-scale transformer feature extraction method fused with channel attention

**Figure 2:**
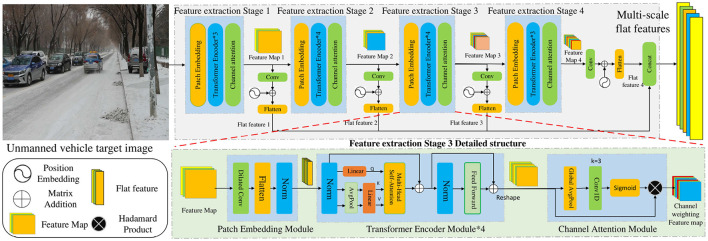
Multi-scale transformer feature extraction method fused with channel attention.

The CNN-based feature extraction method is limited by the receptive field, which makes it difficult to fully extract the feature information of multi-scale change objects, resulting in inaccurate detection. Therefore, we adopt the multi-scale Transformer feature extraction method fused with channel attention to obtain the feature information of multi-scale change objects, as shown in Figure 2. The method consists of four feature extraction stages, each of which contains three modules: a patch-embedding module, a Transformer encoder module, and a channel attention module. Through feature extraction at different stages, feature maps 1–4 are obtained, respectively. The channel dimension transformation, position encoding, flattening, and other operations were performed on the feature maps 1–4 to obtain the flat feature maps 1–4. Finally, the four flat features were fused and spliced to obtain the multi-scale flat features. Compared with the original CNN-based network, the traditional convolution is replaced by dilated convolution in the patch embedding module, which enhances the connection between adjacent patch blocks and extracts more local continuous information. The global receptive field is obtained by introducing the Transformer encoder module to promote the global modeling capability of the network. The channel attention module is adopted to weight the channel features and extract more channel feature information. Multi-scale flat feature fusion is adopted to obtain multi-scale feature information. Therefore, the research content includes a patch embedding module, a Transformer encoder module, a channel attention module, and multi-scale flat feature fusion.

#### 3.1.1 Patch embedding module

The patch embedding module is primarily used for image scale reduction and flattening. A dilated convolution layer is adopted to replace the original convolution layer, which enhances the direct continuity of different patches. First, the size of the feature map $F_{i}^{2d}\in R^{H\times W\times C}$ of stage *i* is reduced from *H* × *W* to $\frac{H\times W}{P_{i}^{2}}$ (*P*_*i*_ is the number of patches of stage *i*) by a down-sampling operation with the dilated convolutional layer. Subsequently, it enters the flattened layer, $F_{i}^{2d}$ is converted into a flat feature $F_{i}^{1d}$, and the normalization operation is performed. Finally, the flat feature is output. The specific operation is as shown in Equation (1):


(1) $$\begin{array}{l} F_{i}^{1d}=Norm\left(Flatten\left(DConv\left(F_{i}^{2d}\right)\right)\right) \end{array}$$

where *DConv* represents a dilated convolution block with a convolution kernel size *P*_*i*_, step size *P*_*i*_/2, and dilated rate of 2. The flattened layer is responsible for converting the feature map from two- to one-dimensional features. *Norm* is the normalization layer, which prevents gradient explosion. After image reduction operations of different stages of patch embedding modules, feature maps of different scales can be obtained.

#### 3.1.2 Transformer encoder module

The Transformer encoder layer at each stage contains *L*_*i*_ layer encoders and each layer encoder is mainly composed of a multi-head self-attention module and feedforward layer. The multi-head self-attention module is adopted to calculate the feature vector (*Q, K, V*) to extract features. When generating *K* and *V*, the AvgPool layer is used for the down-sampling operation, which is implemented as follows [the specific operations are shown in Equations (2–4)]:


(2) $$\begin{array}{l} Q=Linear\left(F_{i}\right) \end{array}$$

(3) $$\begin{array}{l} \;K,V=Linear\left(Reshape2\left(AvgPool\left(Reshape1\left(F_{i}\right)\right)\right)\right) \end{array}$$

(4) $$\begin{array}{l} Attention\left(Q,K,V\right)=Softmax\left(\frac{Q\cdot K^{T}}{\sqrt{d_{head}}}\right)V \end{array}$$

where *Q, K, V* are the three vectors required in the attention calculation: the query, key, and value, respectively. *AvgPool* operations are used to down-sample *K* and *V* such that their dimensions are changed from (*H* × *W, C*) to $\;\left(\frac{H\times W}{R^{2}},C\right)$, *R* = 2. *Reshape*1 can convert one-dimensional features into two-dimensional features, and *Reshape*2 can convert two-dimensional features into one-dimensional features. *Linear* represents a linear mapping operation that converts one-dimensional features into vectors. After feature extraction by the multi-layer Transformer encoder, flat features are output.

#### 3.1.3 Channel attention module

The channel attention module weights the feature map by channel to avoid losing essential channel information in the subsequent dimension transformation process. First, the flat features *F*_*i*_ extracted by the Transformer encoder are transformed into 2-D features using the reshape operation. Subsequently, the dimension of *F*_*i*_ is converted to 1 × 1 × *C* by global average pooling, and the local cross-channel method is used to obtain the information weight of adjacent channels. The weight calculation formula is given by Equations (5, 6):


(5) $$\begin{array}{l} k=\frac{\log_{2}\left(C\right)}{\gamma }+\frac{b}{\gamma } \end{array}$$

(6) $$\begin{array}{l} w_{i}=\sigma \left(\overset{k}{\underset{j=1}{\sum }}w_{i}^{j}y_{i}^{j}\right),y_{i}^{j}\in \Omega_{i}^{k} \end{array}$$

where *k* represents the size of the convolution kernel, *C* represents the channel dimension of the feature map, *b* and γ are hyperparameters, set as *b* = 1, γ =2, $\;y_{i}^{j}$ is the feature of the *j*-th nearest neighbor channel of the *i*-th channel, $\;w_{i}^{j}$ represents the weight of channel $\;y_{i}^{j}$, $\;\Omega_{i}^{k}$ indicates the set of *k* adjacent channels of $\;y_{i}^{j}$, σ represents the sigmoid function, and the specific implementation process given by Equation (7):


(7) $$\begin{array}{l} \hat{F_{i}}=\left(\sigma \left(Conv1D_{k}\left(F_{i}\right)\right)\right)⊗F_{i} \end{array}$$

where *F*_*i*_ is the input feature map, $\;\hat{F_{i}}\in R^{H\times W\times C}$ is the channel weighted feature map, and *Conv*1*D* is a one-dimensional convolutional layer, ⊗ represents matrix multiply.

#### 3.1.4 Multi-scale flat feature fusion

First, $\;\hat{F_{i}}$ is input into the convolutional layer for channel dimension transformation, followed by position embedding and input into the flattening layer for flattening operation. Finally, the multi-scale flat features are fused by Equations (8, 9):


(8) $$\begin{array}{l} PE_{\left(x,2j\right)}^{i}=\sin(\frac{x}{T^{\frac{2j}{d}}}),PE_{\left(x,2j+1\right)}^{i}=\cos(\frac{x}{T^{\frac{2j}{d}}}) \end{array}$$

(9) $$\begin{array}{l} Token=Concat(Flatten(PE^{i}\text{+}Conv(\hat{F_{i}}))) \end{array}$$

where *PE*^*i*^ stands for the position embedding of stage *i*, 2*j* and 2*j* + 1 denote the indices in the encoded vectors, *T* is a constant (the default is 20), *d* is the channel number of the feature map, and *x* is a float between 0 and 1 representing bounding box coordinates (Liu S. et al., 2022). $\;\hat{F_{i}}$ is the output feature map of stage *i*, *Flatten* and *Concat* represent flattening and fusion operations, respectively, and *Token* represents the multi-scale flat feature map.

### 3.2 Training method for query denoising with Gaussian decay

**Figure 3:**
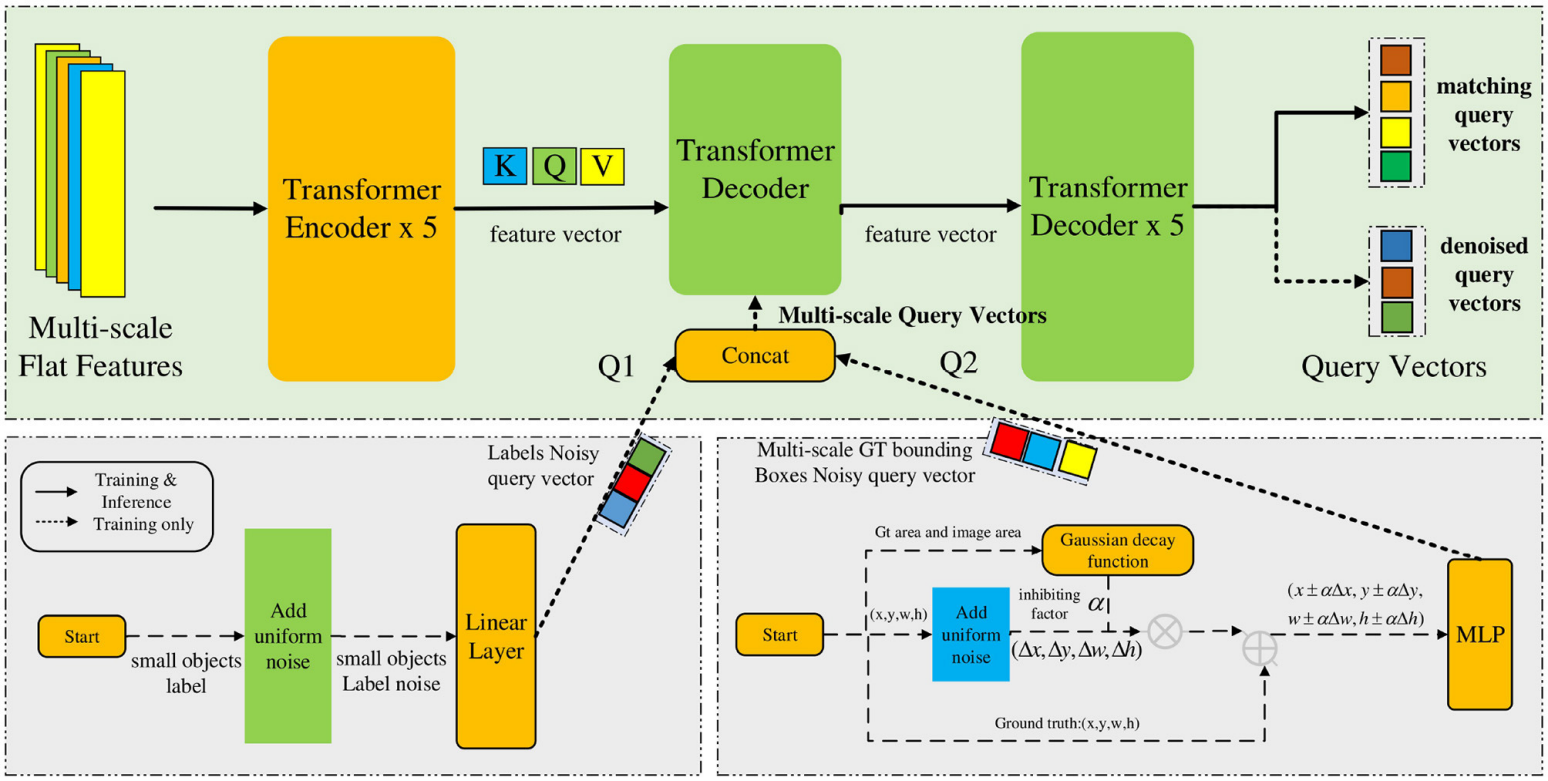
Training method for query denoising with Gaussian decay.

Because of the low resolution and complexity of unmanned images, the feature information contained in the images is insufficient, which leads to low accuracy of small object detection. To improve the training convergence speed and accuracy, DN-DETR introduced a query denoising training method (Li F. et al., 2022). Inspired by this method, this study introduces a training method for query denoising based on Gaussian decay to solve the problem, as shown in Figure 3. The multi-scale flat features are input into the Transformer encoder to obtain feature vectors (*Q, K, V*). The small object label noise is generated by a uniform distribution, and the Gaussian decay function is used to construct GT bounding box noise, which is then converted into multiscale query vectors that are input into the Transformer decoder together with the feature vector for training the output query vector. DN-DETR believes that the instability of bipartite graph matching leads to slow convergence of the model; therefore, it proposes a query-denoising training method to enhance the stability of Hungarian matching. However, this method does not consider the scale difference of the objects; therefore, the detection performance for small objects is not significantly enhanced. The proposed method adds a large degree of noise to a small object to learn more positive and negative sample features of small objects in the process of noise reduction and strengthens the learning for small targets. Therefore, the specific content includes small object label noise addition, multi-scale ground truth box noise addition, and a training method for multi-scale query denoising.

#### 3.2.1 Small object label noise addition

First, a small object is identified according to the ground truth (*gt*) box area of the object, and a false category label is generated by a uniform distribution to replace the real category of the small object to generate a small object label noise. Then, the noise is converted into a small object-label noisy query vector by linear mapping operation. This operation is shown in Equation (10):


(10) $$\begin{array}{l} query\text{\_}label_{k}=Linear\left(\delta \left(target_{m},noise\text{\_}label_{m}\right)\right),k=1,2,...n \end{array}$$

where *query*_*label*_*k*_ is the k-th group label noisy query vector, *target*_*m*_ is the true label of the m-th object, *noise*_*label*_*m*_ is the false label of the m-th object, σ represents the substitution operation, *Linear* represents the linear mapping, which can convert the label noise into a query vector, and *n* is the number of groups of the noisy query vector.

#### 3.2.2 Multi-scale ground truth box noise addition

**Figure 4:**
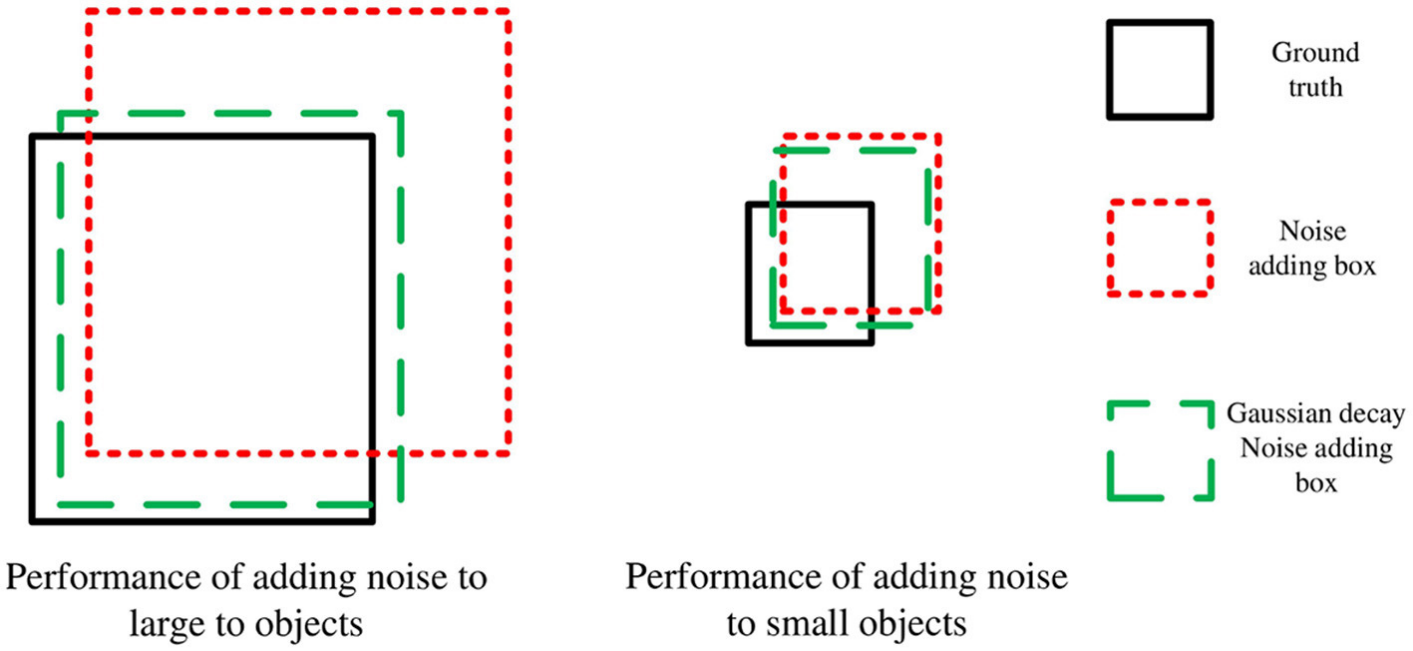
Performance of adding noise to objects of different scales.

Noise is then added to the size and position of the ground truth (gt) box, and the gt box is known to be (*x, y, w, h*). Firstly, the offset threshold coefficient ω_1_ and scaling threshold coefficient ω_2_ are selected in (0,1), and the uniform distribution is used to select the offset coefficient λ_1_ in (0, ω_1_), and the scaling coefficient λ_2_ is selected in (0, ω_2_). Then, we distinguish objects of different scales according to the area of the gt box of the object and use the Gaussian decay function to add different degrees of noise to objects of different scales. The specific method is to generate the inhibition factor α with the Gaussian decay function, and the final noisy box is generated according to Equation (11):


(11) $$\begin{array}{l} noise\text{\_}box=\left(x\pm \alpha \Delta x,y\pm \alpha \Delta y,w\pm \alpha \Delta w,h\pm \alpha \Delta h\right) \end{array}$$

where α = *a*exp(−(*area* − *b*)^2^/2*c*^2^), *area* represents the ratio of the area of the image to the area of the gt box, *b* represents the offset value used to reduce the difference between the objects, 2*c*^2^ represents the degree of suppression (the smaller 2*c*^2^ is, the more obvious the suppression performance is), and $\;\Delta x=\frac{\lambda_{1}w}{2}$, $\;\Delta y=\frac{\lambda_{1}h}{2}$, Δ*w* = λ_2_*w*, Δ*h* = λ_2_*h*, which ensures $\;|\Delta x|<\frac{\omega_{1}w}{2}$, $\;|\Delta y|<\frac{\omega_{1}h}{2}$, Δ*w* ∈ [(1−ω_2_)*w*, (1+ω_2_)*w*], Δ*h* ∈ [(1−ω_2_)*h*, (1+ω_2_)*h*]. The noise addition performance of different scale objects is shown in Figure 4. As shown in Figure 4, after Gaussian decay suppression, the position offset and size scaling performances of small objects are unchanged, while those of large objects are significantly inhibited, which means that the model will obtain more positive and negative sample information of small objects, thereby strengthening the learning of the model for small objects. The multi-scale gt box noise is then converted into a multi-scale gt box noisy query vector through the MLP layer, as shown in Equation (12):


(12) $$\begin{array}{l} query\text{\_}box_{k}=MLP\left(noise\text{\_}box_{m}\right),k=1,2,...n \end{array}$$

where *query*_*box*_*k*_ is the *k*-th group of gt box noisy query vectors, *MLP* is the mapping network composed of three linear layers, α is the inhibitory factor generated by Gaussian decay function, and *noise*_*box*_*m*_ is the noisy gt box of the *m*-th object.

#### 3.2.3 Training method for multi-scale query denoising

The generated small object label noisy query vector and multi-scale gt box noisy query vector are concatenated to constitute a multi-scale query vector, as shown in Equations (13–15):


(13) $$\begin{array}{l} Q_{1}=Concat\left(query\text{\_}label_{k}\right),k=1,2,...n \end{array}$$

(14) $$\begin{array}{l} Q_{2}=Concat\left(query\text{\_}box_{k}\right),k=1,2,...n \end{array}$$

(15) $$\begin{array}{l} Q_{\text{ms}}=Concat\left(Q_{1},Q_{2}\right) \end{array}$$

where *Q*_1_ represents the multi-scale label noisy query vector, *Q*_2_ represents the multi-scale gt box noisy query vector, *Q*_ms_ represents the final generated multi-scale query vector, and *Concat* represents the concatenation operation. Finally, *Q*_ms_ is input into the Transformer decoder together with the outputs *K, Q, V* of the Transformer encoder to output the query vector. The query vector is divided into a matching query vector and a denoised query vector. The denoised query vector is acquired from the noisy query vector after denoising training. As the generated query vector contains real object information, the denoised query vector does not require a matching operation, and the loss can be calculated directly to update the network parameters.

In this study, the experiments showed that by adding a large degree of noise to small objects, the detection accuracy of objects of other scales will slightly decrease, but the detection accuracy of small objects will significantly increase. This is because the method improves the difficulty of small objects' noise recovery and provides the model with more positive and negative sample information on small objects, thus strengthening the model's learning ability for small objects.

### 3.3 Hybrid matching method based on optimal transport and Hungarian

**Figure 5:**
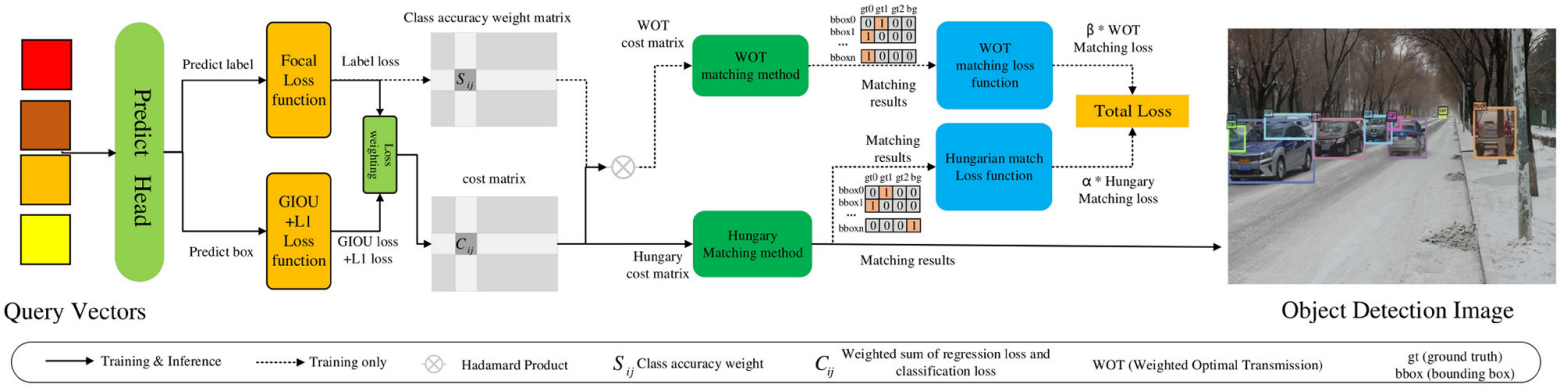
Hybrid matching method based on optimal transport and Hungarian.

In an unmanned driving scene, owing to the occlusion of vehicles and pedestrians, it is difficult to distinguish object features; therefore, there are problems of missed and false detections of densely occluded objects. In this regard, this study adopted a hybrid matching method based on optimal transport and Hungarian to complete the matching process between the ground truth and predicted objects, to expand the number of positive samples and improve the detection performance of densely occluded objects, as shown in Figure 5. First, after decoding the query vector to obtain the prediction box and prediction category, class and regression losses are calculated. Then, the optimal transport and Hungarian cost matrices are generated according to the loss value, and the optimal transport matching method and Hungarian matching method are used to obtain the matching results. Finally, the two matching losses are multiplied by the corresponding weight coefficients α and β to obtain the total loss, which is used to guide the model to update parameters. Meanwhile, the object detection image is output according to the Hungarian matching result. The original method adopts only the Hungarian matching method to match the ground truth and predicted box. Although this one-to-one matching method avoids post-processing operations, such as NMS, most of the prediction boxes are divided into backgrounds, resulting in limited positive sample features. Thus, there are problems of missed and false detections of occluded objects. The hybrid matching method can help the model obtain abundant positive sample features in the process of matching the predicted and ground truth objects to effectively solve this problem. Therefore, the specific content includes the optimal transport matching method branch, Hungarian matching method branch, and hybrid matching method.

#### 3.3.1 Optimal transport matching method branch

As a one-to-many matching method, the optimal transport matching method can be used to solve the matching issue between ground truth objects and predicted boxes. It consists of two main processes: constructing the cost matrix and solving the allocation scheme.

The first is the construction of the cost matrix. For picture *I*, there are *m* ground truth (gt) objects and *n* predicted boxes (pbox), and the cost of matching the i-th *gt* object and j-th *pbox* is *C*_*ij*_. For the cost matrix *C*, if the predicted box *pbox*_*j*_ is a positive sample, the cost of matching *gt*_*i*_ with *pbox*_*j*_ is $\;C_{ij}^{fg}$, where $\;C_{ij}^{fg}$ is the weighted sum of the class loss and regression loss, as shown in Equation (16):


(16) $$\begin{array}{l} C_{ij}^{fg}=\alpha L_{cls}\left(p_{j}^{cls}\left(\theta \right),g_{i}^{cls}\right)+\beta L_{reg}\left(p_{j}^{box}\left(\theta \right),g_{i}^{box}\right) \end{array}$$

where $\;p_{j}^{cls}$, $\;p_{j}^{box}$ denote the class prediction and prediction box localization results, respectively, and $\;g_{i}^{cls}$, $\;g_{i}^{box}$ represent the true class and ground truth box, respectively. *L*_*cls*_ represents the classification loss, as shown in Equation (17). *L*_*reg*_ is regression loss, which is used to measure the difference between the position and size of the pbox and gt box. In this study, GIOU and L1 were adopted to calculate the regression loss of the predicted boxes (Rezatofighi et al., 2019), as shown in Equations (18, 19):


(17) $$\begin{array}{l} L_{cls}=-\alpha \overset{n}{\underset{i=1}{\sum }}{\left(1-p_{i}\right)}^{\gamma }log_{2}\left(p_{i}\right)-\left(1-\alpha \right)\overset{n}{\underset{i=1}{\sum }}p_{i}^{\gamma }log_{2}\left(1-p_{i}\right) \end{array}$$

(18) $$\begin{array}{l} L_{GIOU}=1-\frac{\left(A\cap B\right)}{\left(A\cup B\right)}+\frac{A_{c}-\left(A\cap B\right)}{A_{c}} \end{array}$$

(19) $$\begin{array}{l} L1=\frac{1}{N}\overset{N}{\underset{i=1}{\sum }}|f\left(x_{i}\right)-y_{i}| \end{array}$$

where *p*_*i*_ represents the probability that sample *i* is positive, and α and β are the hyperparameters of the focal loss function (Lin et al., 2017). *A* represents the area of the ground truth box, *B* represents the area of the predicted box, and *A*_*c*_ represents the minimum closure region area between the predicted box and ground truth box (Rezatofighi et al., 2019).

In addition, many predicted boxes are negative samples, so the “background” category is introduced to match the negative samples. Classifying a sample as the background class requires only the calculation of the classification loss, as shown in Equation (20):


(20) $$\begin{array}{l} C_{cls}^{bg}=FocalLoss\left(p_{j}^{cls},\emptyset \right) \end{array}$$

where ∅ represents the background class. The final cost matrix *C* ∈ *R*^(*m*+1)×*n*^ is obtained by concatenating *C*^*bg*^ ∈ *R*^1×*n*^with the last row of *C*^*fg*^ ∈ *R*^*m*×*n*^. However, it is easy to assume that the categories do not match but the positions are close in the early stages of training, which leads to transmission errors and causes false detection. To avoid the error transmission problem as much as possible, the cost matrix is weighted by class accuracy weight. Class accuracy weight *S* is defined as Equation (21):


(21) $$\begin{array}{l} p_{j}=\frac{exp\left(-z_{j}\right)}{\overset{k}{\underset{i=1}{\sum }}exp\left(-z_{i}\right)},S_{ij}=L_{cls}\left(p_{j},g_{i}^{cls}\right) \end{array}$$

where *p*_*j*_ represents the probability that a predicted box belongs to the *j*-th class, *z*_*i*_ represents the value of the output *i*-th pbox, and *S*_*ij*_ represents the probability that the *i*-th pbox belongs to the *j*-th true class. The construction formula for the weighted optimal transport cost matrix *C* is given by Equation (22):


(22) $$\begin{array}{l} C={\left(C⊙S\right)}_{ij} \end{array}$$

where *S* represents the class accuracy weight matrix and *C*_*ij*_ is the cost loss value between the *i*-th pbox and *j*-th gt box, ⊙ represents Hadamard product.

This is followed by solving the allocation plan. The objective function of the optimal transport matching is to obtain an allocation plan $\;\pi^{*}\in R^{\left(m+1\right)\times n}=\left\{\pi_{ij}|i=1,2,...m+1,j=1,2,...n\right\}$, as shown in Equation (23):


(23) $$\begin{array}{r} \underset{\pi }{min}\sum_{i=1}^{m+1}\sum_{j=1}^{n}C_{ij}\pi_{ij},(\sum_{i=1}^{m}\pi_{ij}=d_{j},\sum_{j=1}^{n}\pi_{ij}=s_{i},\sum_{i=1}^{m+1}s_{i}=\sum_{j=1}^{n}d_{j},\\ \pi_{ij}\geq 0,i=1,2,...,m+1,j=1,2,...,n) \end{array}$$

(24) $$\begin{array}{l} s_{i}={\{}_{n-m\times k,i=m+1}^{k,i\leq m} \end{array}$$

where *s*_*i*_ is the number of predicted boxes matched by the *i*-th ground truth box [as shown in Equation (24)], and *d*_*j*_(*d*_*j*_ = 1) is the number of ground truth boxes matched by the *j*-th predicted box. Then, we use the dynamic *top* − *k* strategy instead of the Sinkhorn-Knopp algorithm to obtain the optimal transport matching scheme π^*^, which is the allocation matrix of the predicted and ground truth boxes.

#### 3.3.2 Hungarian matching method branch

In image *I*, there are *m* ground truth(gt) and *n* predicted objects, and assuming *n* > *m*, the number of gt objects is extended to *n* by padding ∅, where ∅ represent the background class. The construction process of the cost matrix is the same as that of the optimal transport matching method. The objective function of Hungarian matching aims to obtain an allocation plan $\;u\in R^{n\times n}=\left\{u_{ij}|i=1,2,...n,j=1,2,...n\right\}$, as shown in Equation (25):


(25) $$\begin{array}{r} \underset{u}{min}\sum_{i=1}^{n}\sum_{j=1}^{n}C_{ij}\mu_{ij},(\sum_{i=1}^{n}\mu_{ij}=1,\sum_{j=1}^{n}\mu_{ij}=1,\sum_{i=1}^{n}\sum_{j=1}^{n}\mu_{ij}=n,\\ \mu_{ij}\geq 0,i=1,2,...,n,j=1,2,...,n) \end{array}$$

where *C* ∈ *R*^*n*×*n*^ is obtained by concatenating *C*^*bg*^ ∈ *R*^(*n*−*m*)×*n*^ with the last row of *C*^*fg*^ ∈ *R*^*m*×*n*^. Then, the cost matrix *C* is input into the Hungarian matching method to obtain the allocation scheme μ of Hungarian matching. Considering that this method is essentially an assignment problem, the linear-sum-assignment function is called directly in this study.

#### 3.3.3 Hybrid matching method

**Figure 6:**
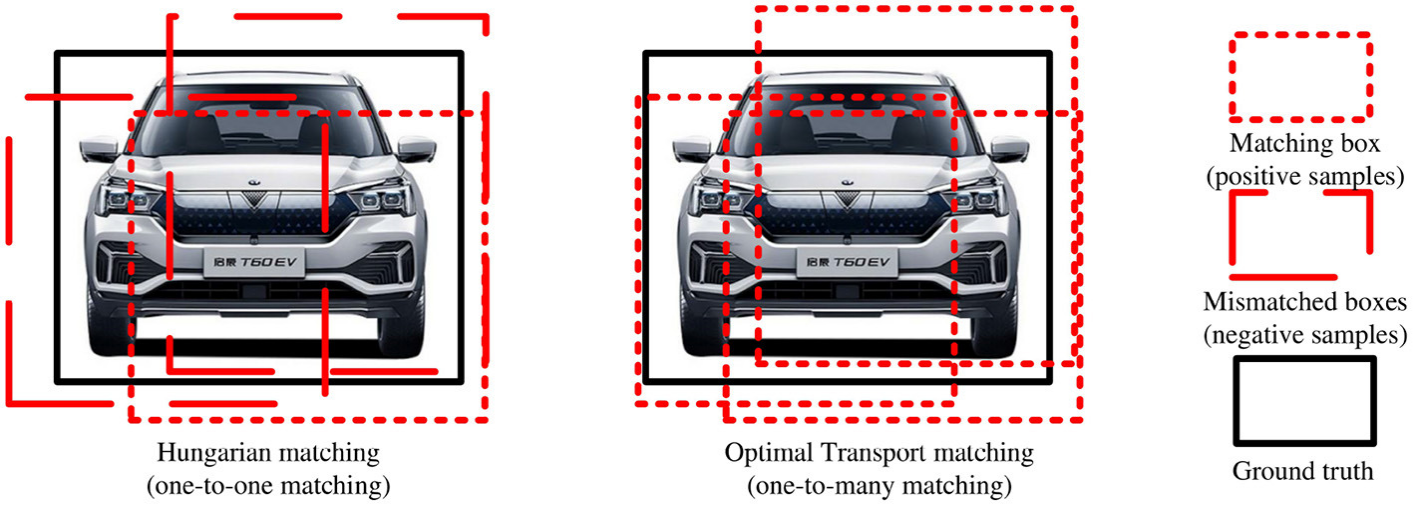
Comparison of matching results between Hungarian matching and optimal transport matching.

The allocation scheme μ of the Hungarian matching method and the matching scheme π^*^ of the optimal transport matching method are adopted to calculate the classification and regression losses, respectively, as shown in Equation (26), and the hybrid matching loss function is shown in Equation (27):


(26) $$\begin{array}{l} \tau_{O},\tau_{H}=\overset{n}{\underset{i=1}{\sum }}\left[L_{cls}\left(p_{\sigma \left(i\right)}^{cls},g_{\sigma \left(i\right)}^{cls}\right)+1_{\left\{g_{\sigma \left(i\right)}^{cls}\neq \emptyset \right\}}L_{reg}\left(p_{\sigma \left(i\right)}^{box},g_{\sigma \left(i\right)}^{box}\right)\right] \end{array}$$

(27) $$\begin{array}{l} \tau =\alpha *\tau_{H}+\beta *\tau_{O} \end{array}$$

where *L*_*cls*_ and *L*_*reg*_ are given in Equations (14–16), σ(*i*) represents the index of the ground truth box corresponding to the i-th predicted box, σ(*i*)is the total loss, τ_*H*_ represents the Hungarian matching loss, τ_*O*_ is the optimal transport matching loss, and α, β are the corresponding loss weights. The training direction of the model is controlled by α and β. As shown in Figure 6, the optimal transport matching method assigns more positive samples to each object, so the model obtains more positive sample features and resolves the issue of missed and false detections of densely occluded objects. This hybrid matching method is only adopted in the training phase; in the inference phase, only the Hungarian matching method is used, thus avoiding post-processing operations, such as NMS. The pseudo-code of the hybrid matching method based on optimal transport and Hungarian is shown in Algorithm 1.

**Algorithm 1:** Hybrid matching method.

**Input:** *I* represents an image, *gt* represents the ground truth in image *I*
**Output:** τ represents total loss
1: *m* ← |*gt*|
2: *P*^*cls*^, *P*^*box*^ = *Forward*(*I, gt*)
3: $\;P^{class}=softmax\left(-P^{cls}\right),S_{ij}=FocalLoss\left(p_{j}^{class},gt_{i}^{cls}\right)$
4: $\;c_{cls}^{ij}=FocalLoss\left(p_{j}^{cls},gt_{i}^{cls}\right),c_{reg}^{ij}=GIOU\left(p_{j}^{box},gt_{i}^{box}\right)$
5: $\;c_{cls}^{bg}=FocalLoss\left(p_{j}^{cls},\emptyset \right),c^{fg}=\alpha c_{cls}+\beta c_{reg}$
6: $\;C=concat\left(c_{cls}^{bg},c^{fg}\right)$
7: *C* = C*S
8: s_*i*_(*i* = 1, 2, ...*m*) ← Dynamic *k* Estimation according to *gt*
9: **for** *i* = 1 to *m* **do**
10: *j* = *topk*(*C*_*i*_, *k* = s_*i*_), *M*_*ij*_ = *true*
11: **end for**
12: **if** *sum*(*M*_*i*_ > 0) > 0 **then**
13: *indices* ← *Filter*(*M*_*i*_)
14: **end if**
15: compute optimal transport matching plan π^*^ from indices
16: *indices* ← *linear*_*sum*_*assignment*(*C*)
17: compute Hungarian matching plan μ from indices
18: $\;\tau_{O}=loss\left(gt,\pi^{*},P^{cls},P^{box}\right),\tau_{H}=loss\left(gt,\mu ,P^{cls},P^{box}\right)$
19: τ = α*τ_*H*_ + β*τ_*O*_
20: **return** τ

### 3.4 Summary

From the above discussion, we propose an improved object detection method for unmanned driving based on Transformers. First, this method uses a multi-scale Transformer feature extraction method fused with channel attention to solve the issue of unsatisfactory multi-scale change object detection performance. Then, a training method for query denoising with Gaussian decay is adopted to solve the low detection accuracy of small objects, and a hybrid matching method based on optimal transport and Hungarian is used to address the issue of missed and false detections of densely occluded objects. In the following section, the improved content of this study is applied to the DINO model, and relevant experiments and analyses are reported.

## 4 Experimental results and analysis

### 4.1 Experimental setup

The experimental dataset for the DETR series models was mostly COCO2017 (Lin et al., 2014). As this study focused on object detection in unmanned driving scenarios, cars, trucks, and buses were selected from the COCO2017 dataset to form a new dataset called COCO-driving. In total, there were 43,250 car objects, 9,096 truck objects, and 6,035 bus objects in this dataset. In addition, this study also uses WiderPerson (Zhang et al., 2019), KITTI (Geiger et al., 2013), and Waymo open (Sun et al., 2020) datasets to conduct experiments to show the advancement of the proposed method. Considering that the Waymo Open dataset is substantial, 15,990 images are selected as the training set and 3,400 images are selected as the validation set. The optimizer used the AdamW optimization algorithm based on weight decay, where batch size = 2 and the initial learning rate was set to 0.00005 on an NVIDIA V100 GPU.

These three improvements were applied to the DINO model to verify the performance of the proposed method, and Average Precision (AP) and Params were used as evaluation indicators. AP was adopted to measure the model detection accuracy, which is the area under the PR (precision-recall) curve. Precision (P) and Recall (R) were calculated using Equations (28, 29):


(28) $$\begin{array}{l} P=\frac{TP}{TP+FP} \end{array}$$

(29) $$\begin{array}{l} R=\frac{TP}{TP+FN} \end{array}$$

(30) $$\begin{array}{l} AP=\int_{0}^{1}P\left(R\right)dR\; \end{array}$$

where TP represents correctly identified positive samples, FP represents incorrectly identified positive samples, and FN represents incorrectly identified negative samples. The mAP is obtained by averaging the AP of multiple categories as shown in Equation (30); the larger the mAP, the higher the detection accuracy, as shown in Equation (31):


(31) $$\begin{array}{l} mAP=\frac{\overset{n}{\underset{i=1}{\sum }}AP_{i}}{n} \end{array}$$

where *AP*_*i*_ is the AP of the *i*-th category, and the IOU thresholds of AP are 0.5 and 0.5 ~ 0.95, which are denoted as mAP_50_ and mAP, respectively. mAP_*s*_ is used to measure the detection performance for small objects of less than 32 × 32 pixels. In addition, The model size is measured using the number of model parameters (Params). GFLOPs are used to measure the computational complexity of the model, and FPS is used to measure the detection speed.

### 4.2 Analysis of experimental parameters

#### 4.2.1 Parametric analysis of the number of channel attention modules

To explore the performance of the number of channel attention modules in the multi-scale Transformer feature extraction method fused with channel attention, this study set the number of channel attention modules *k* as the experimental parameter and designed five groups of experiments with *k* = 0, 1, 2, 3, and 4, where *k* = 0 means that the channel attention module was not used, *k* = 1 means that the channel attention module was used in stage 4, *k* = 2 means that the channel attention module was used in stages 3 and 4, and so on. Because multi-scale objects are to be studied, the mAPs of all objects (mAP-all), medium objects (mAP-medium), and small objects (mAP-small) were compared.

**Figure 7:**
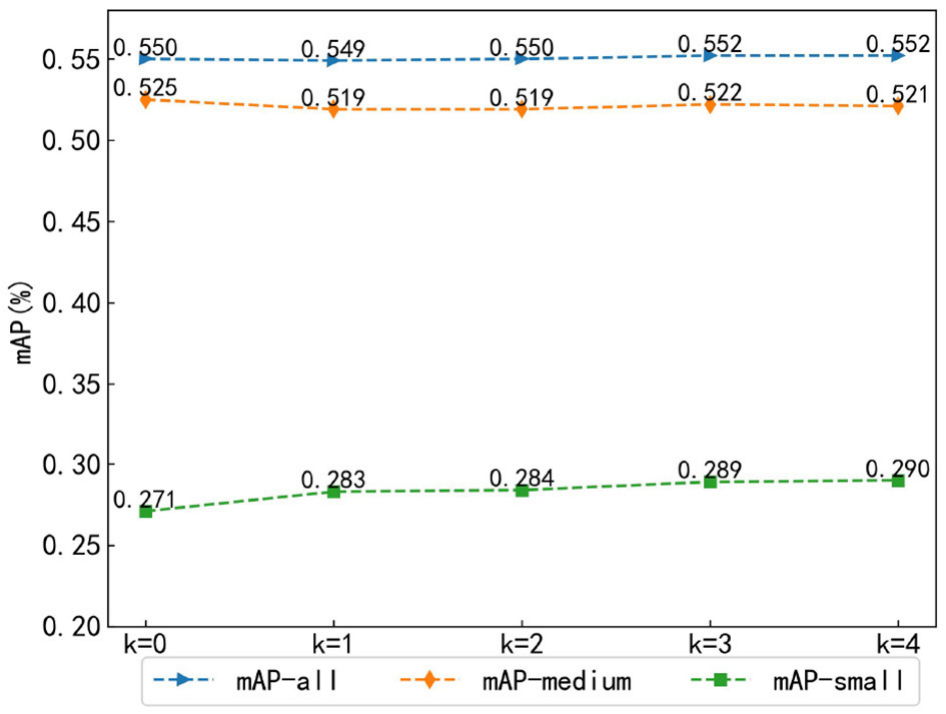
Parametric analysis of the number of channel attention modules.

As shown in Figure 7, in the mAP-all comparison, it has a slight upward trend with the increase of *k* and finally achieves the maximum value at *k* = 4. However, in the mAP-medium comparison, it shows a slow decreasing trend with the increase of *k*, while in the mAP-small comparison, it presents a clear increasing trend with the increase of *k*, again reaching a maximum value at *k* = 4. As the information of small targets is preserved completely in the low-level features of the image, the detection effect of small objects gradually becomes better with the increase of the number of channel attention layers. In unmanned driving scenarios, the detection of small objects becomes challenging; hence, a slight drop in mAP-medium is acceptable.

#### 4.2.2 Parameter analysis of the training method for query denoising with Gaussian decay

To explore the suppression performance of the Gaussian decay function with different parameters on objects of different scales, we analyzed the *k, a*, and *c* parameters of *f*(*x*) = *a*exp(−(*area* − *b*)^2^/2*c*^2^). For convenient representation, *k* = 2*c*^2^ was set, *a* = 1, *b* = 0, *k* = 0.005, 0.01, 0.05, 0.1 and *a* = 1, *b* = 0.01, *k* = 0.005, 0.01, 0.05, 0.1, for a total of eight groups of experiments, and mAP-all and mAP-small were compared.

**Figure 8:**
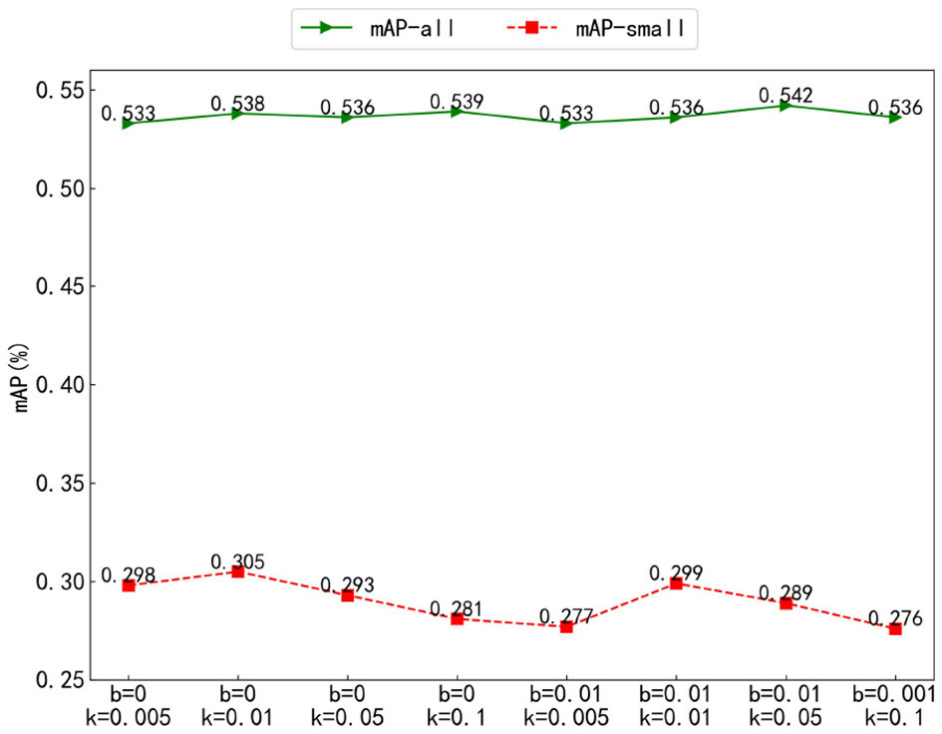
Parameter analysis of the training method for query denoising with Gaussian decay.

As shown in Figure 8, in the mAP-all comparison, the results of the eight groups of experiments are quite similar. However, in the comparison of mAP-small, when the *b* value is the same, the overall trend increases first and then decreases with the increase of *k*. For the same value of *k*, the results with *b* = 0 are all higher than those with *b* = 0.01. The best result is obtained when *b* = 0 and *k* = 0.01. This is because *k* can control the width of the Gaussian figure, thus affecting the scaling performance of objects of different scales. When the value of *k* is too small, it will significantly enhance the suppression performance between small objects, resulting in a large gap between small objects and a slight decline in the detection performance of small objects. When *k* is too large, there will be no obvious size difference between different objects, and the suppression performance of the Gaussian decay function will fail. *b*, as the offset coefficient, can shift the Gaussian figure to reduce the scale difference between objects. When *b* = 0, the difference between different scale objects is obvious, and when *b* = 0.01, the difference between different scale objects becomes small, which affects the suppression performance and leads to a decline in detection accuracy.

#### 4.2.3 Weight parameter analysis of hybrid matching method

**Figure 9:**
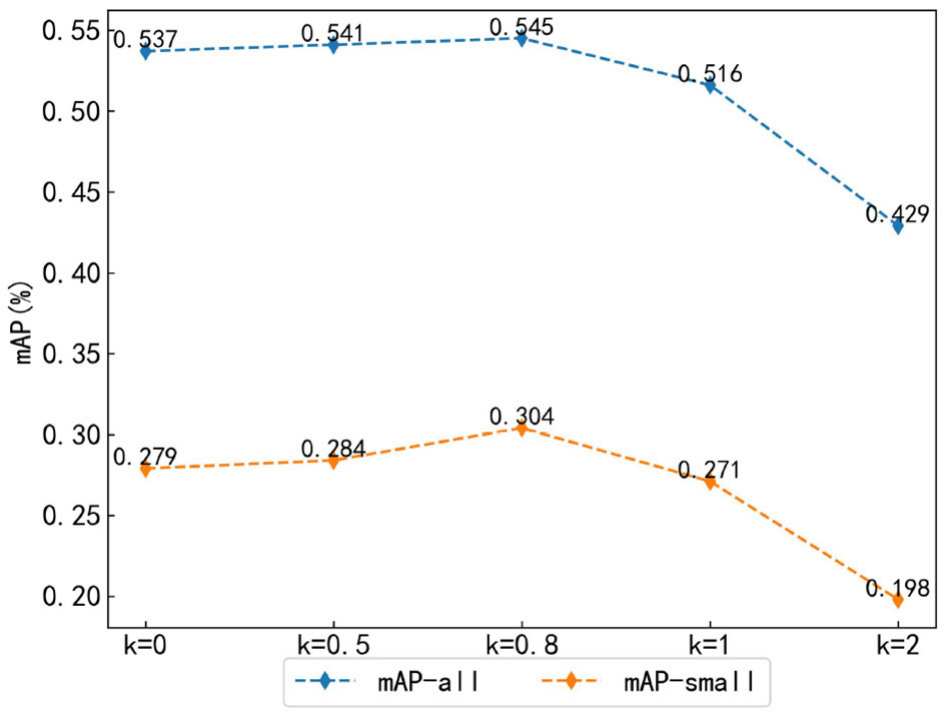
Weight parameter analysis of hybrid matching method.

To explore the influence of weight parameters on the results of the hybrid matching method based on optimal transport and Hungarian, this study adjusted the weight parameter *k* of optimal transport matching to different values: *k* = 0, 0.5, 0.8, 1, 2. Here, *k* = 0 indicates that the optimal transport matching method is not used, while the weight parameter α of Hungarian matching remains constant at 1. By adjusting the weight parameters, the balance between the two matching methods' influence can be controlled during the model training. The evaluation metrics in this study were mAP-all and mAP-small. As shown in Figure 9, according to the changes of mAP-all and mAP-small, both of them show a trend of first increasing and then decreasing as *k* increases. Among them, when *k* = 0.8, the results reach the highest value, mAP-all is 0.545, and mAP-small is 0.304, which are 0.8 and 2.5% higher than that of the original method (*k* = 0). This is because as a one-to-many matching strategy, the optimal transport matching can obtain more positive sample features and promote the detection accuracy of the collaborative model when the weight proportion is small. However, when the weight proportion is large, it will mislead the Hungarian matching process, thus dominating the training direction of the model, and leading to a decrease in detection accuracy.

### 4.3 Ablation studies and analysis

To evaluate the performance of the proposed method, we applied the three improvements to the DINO model and conducted ablation experiments on the COCO-driving dataset, as shown in Table 1, Exp1 represents the original method. Exp2 incorporates a multi-scale Transformer feature extraction method fused with channel attention, exhibiting improved small object detection performance with a 1.4% increase in mAP and a 1.5% increased in mAP_*s*_. Exp3 involves training with query denoising using Gaussian decay, resulting in a modest 0.1% increase in mAP but a notable 2.4% improvement in small object features. Exp4 utilizes a hybrid matching method of optimal transport and Hungarian, leading to a 0.6% increase in mAP and a significant 2.5% increase in mAP_*s*_, demonstrating effectiveness in occlusion of dense occlusion object detection owing to fewer pixels being available. Thus, Exp4 shows that the proposed method is ideal for dense occlusion object detection. Exp5 is the experimental result of applying three improved contents together. Compared with the original method, mAP increases by 1.5% and mAP_*s*_ by 2.3%. Although mAP and are not as good as some methods, for example, mAP decreases by 0.2% compared with Exp2, mAP_*s*_ increases by 0.7%. In addition, from the perspective of the AP of each category, the APs of Exp1 are 0.484, 0.420, and 0.707, respectively, and those of Exp5 are 0.502, 0.433, and 0.718, respectively. The detection accuracy of each category in other experimental groups also improved to different degrees. In the measurement process of parameters and computation, the uniform image size is 640 × 640. From the perspective of Params, compared with the original method, the proposed method is reduced by 7.89 From the results of model computational complexity (GFLOPs) and model processing speed (FPS), the results of Exp1 are mirror those of Exp3 and Exp4. This is because the innovative content of Exp3 and Exp4 is only enabled in the training phase, and the Transformer feature extraction network fused with channel attention is adopted in Exp2 and Exp5. Compared with the original method, the GFLOPs of Exp2 and Experiment 5 increased by 38%, and the FPS decreased by 21%. It is evident that the real-time performance of the proposed method is poor, and the FPS is 18, which cannot meet the real-time requirements. However, the poor real-time performance of the proposed method is caused by the high computational complexity of the Transformer feature extraction network that fuses channel attention, and the other two improvements will not affect the real-time performance of the model, so we can consider reducing the feature extraction network to improve the real-time performance of the model.

**Table 1:** Experimental results on the COCO-driving.

**ID**	**MTC**	**GDN**	**HM**	**mAP**	** mAP_*s*_**	** AP_*car*_**	** AP_*truck*_**	** AP_*bus*_**	**Params**	**GFLOPs**	**FPS**
Exp1				0.537	0.279	0.484	0.420	0.707	46.67M	279	23
Exp2	✓			0.551	0.294	0.510	0.428	0.719	38.78M	284	18
Exp3		✓		0.538	0.305	0.482	0.432	0.714	46.67M	279	23
Exp4			✓	0.545	0.302	0.490	0.434	0.710	46.67M	279	23
Exp5	✓	✓	✓	0.552	0.304	0.502	0.433	0.718	38.78M	284	18

We use the terms “MTC,” “GDN,” and “HM" to denote “Multi-scale Transformer with Channel Attention,” “Gaussian decay De-Noising Training,” and “Hybrid Matching,” respectively.

**Figure 10:**
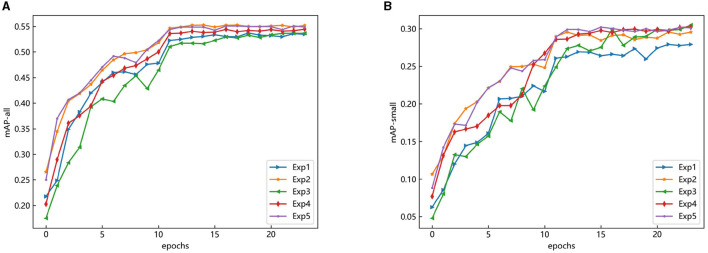
AP convergence change diagram of the proposed method on the COCO-driving dataset. **(A)** mAP of all objects. **(B)** mAP of small objects.

The changes in the AP values in the above experiments are shown in Figure 10. The improved content was applied to the DINO model and trained for 24 epochs. From the overall point of view, it shows a sharp rise in the first, then tends to be a flat trend. At the 12th epoch, the mAP (map-all) and mAP_*s*_ (mAP-small) value can be seen to improve rapidly, which is because the dropout operation is used to remove redundant parameters and avoid overfitting so that the performance of the proposed method on the validation set can be improved. In addition, it can be seen that the curve of mAP-small fluctuated greatly, which was caused by using mAP-all as the evaluation criterion to update the network parameters during the training process, and the small object features were difficult to learn.

**Figure 11:**
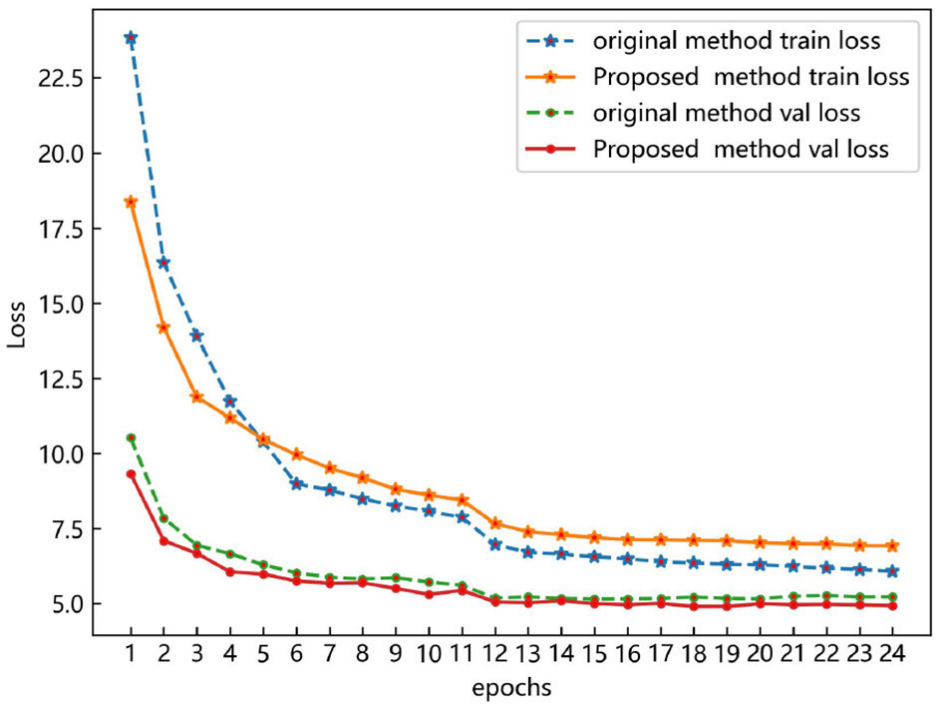
Loss curves of the proposed method and the original method on the COCO-driving dataset.

In Figure 11, both the proposed method and the original method exhibit a sharp decrease in loss value initially, followed by gradual convergence and eventual stabilization at the 20th epoch. Despite incorporating optimal transport matching loss into the hybrid matching method during training, the proposed method maintains a lower training loss than the original method, indicating superior feature learning capability. Additionally, the validation loss of the proposed method is slightly lower than that of the original method, indicating the absence of overfitting and validating the proposed method's performance.

### 4.4 Comparison with state-of-the-art object detection methods

#### 4.4.1 Objective analysis

To verify the superiority of our method over other advanced object detection methods, we conduct comparative experiments, including Transformer-based methods: DAB-DETR (Liu S. et al., 2022), DN-DETR (Li F. et al., 2022), Deformable-DETR (Zhu et al., 2020) and DINO (Zhang et al., 2022). Two-stage methods: Fsater-RCNN (Ren et al., 2015) and Sparse R-CNN (Sun et al., 2021); One-stage methods: YOLOX (Ge et al., 2021b) and YOLOv7 (Wang et al., 2023). These methods are tested on COCO-driving (Lin et al., 2014), WiderPerson (Zhang et al., 2019), KITTI (Geiger et al., 2013), and Waymo Open (Sun et al., 2020) datasets, and the experimental parameters, with consistent environmental experiments. The results are presented in Tables 2–5.

**Table 2:** Experimental results of the proposed method and existing methods on COCO-driving dataset.

**Model**	**mAP_50_**	**mAP**	**mAP_*s*_**	**Epoch**	**Params**	**GFLOPs**	**FPS**
DAB-DETR (Liu S. et al., 2022)	0.672	0.424	0.215	50	43.87M	94	17
DN-DAB-DETR (Li F. et al., 2022)	0.681	0.445	0.183	50	43.47M	101	16
Deformable-DETR (Zhu et al., 2020)	0.626	0.438	0.264	50	39.85M	196	22
DN-Deformable-DETR	0.721	0.487	0.307	50	47.18M	265	23
DN-DAB-Deformable-DETR	0.720	0.533	0.293	50	47.21M	273	15
DINO (Zhang et al., 2022)	0.728	0.537	0.273	24	46.67M	279	23
Faster-RCNN (Ren et al., 2015)	0.621	0.455	0.266	36	41.32M	180	26
Sparse-RCNN (Sun et al., 2021)	0.612	0.428	0.267	36	41.35M	87	23
YOLOX-l (Ge et al., 2021b)	0.721	0.526	0.231	100	54.21M	155	69
YOLOv7 (Wang et al., 2023)	0.747	0.550	0.298	100	36.91M	103	161
Proposed method	0.742	0.552	0.304	24	38.78M	284	18

The experimental results of the proposed method and existing advanced methods on the COCO-driving dataset are shown in Table 2. Compared with Transformer-based object detection methods, the proposed method exhibits notably advantages in detection accuracy and parameter quantity. For example, compared to the original method (DINO), the proposed method achieves an increase of 2.7 and 11% in mAP and mAPs, respectively, while reducing parameter quantity (Params) by 16%. The GFLOPs only increases by 1.7%; however, regarding detection speed (FPS), the FPS of the proposed method decreases by 21%. Additionally, while Deformable-DETR shows a 1% higher mAPs than the proposed method, the latter outperforms in mAP by 13% with 8.4M fewer parameters, making the cost acceptable. Comparing with two-stage object detection surpassing Faster-RCNN and Sparse-RCNN by 21 and 28% in mAP, respectively. Concurrently, the number of parameters of the proposed method is also reduced by 2.54M and 2.57M, respectively. However, from the perspective of GFLOPs, the computational complexity of Sparse-RCNN is the lowest among all methods, and the proposed method is 226% higher than that of the proposed method, which means that the proposed method requires higher hardware performance in the training and inference process. Compared with the single object detection method, the detection accuracy of the proposed method is still ahead. While the mAP_50_ of YOLOv7 is 0.6% higher than that of the proposed method, the mAP_*s*_ of the proposed method is 2% higher than that of YOLOv7, which indicates that the proposed method has a better effect on small object detection. Thus, while the proposed method boasts the highest detection accuracy, its computational complexity and detection speed require improvement. Addressing these concerns will be the focus of future research endeavors.

**Figure 12:**
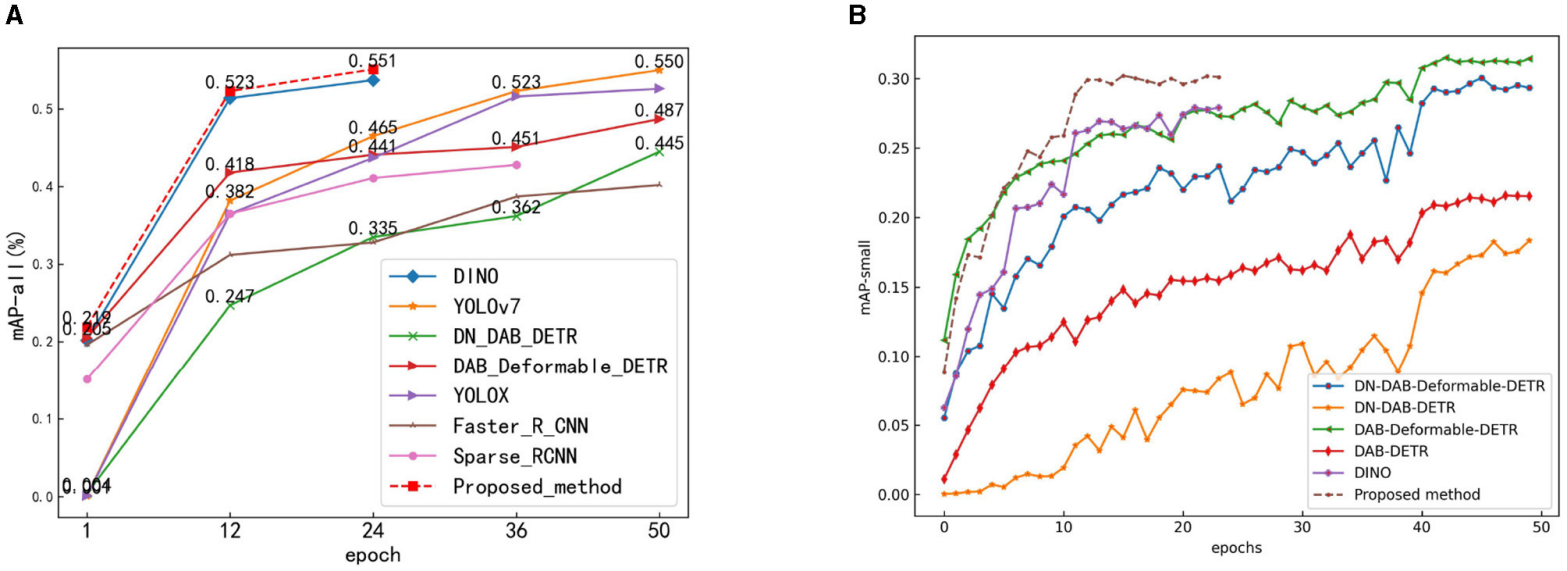
The mAP convergence curves of the method proposed and other object detection methods. **(A)** mAP-all convergence curves. **(B)** mAP-small convergence curves.

The mAP convergence curves of the proposed method and some object detection methods using the COCO-driving dataset are shown in Figure 12. This figure indicates that the proposed method achieved satisfactory results after 24 training epochs. Figure 12A shows that the mAP-all value of the proposed method was 0.552, which is higher than those of the other methods. Figure 12B shows that only DN-DAB-Deformable-DETR is slightly higher in the detection accuracy of small objects than the proposed method. The detection accuracies of the other methods were lower than that of the proposed method. Therefore, on the whole, the detection accuracy of the proposed method is still the highest.

The experimental results of the proposed method and advanced methods on the WiderPerson dataset are illustrated in Table 3. The dataset notably presents densely occluded pedestrians, posing significant challenges for object detection. Despite this, the proposed method maintains a leading position in detection accuracy. Among the transformer-based object methods, DINO achieves the highest detection accuracy. DN-Deformable-DETR, originally excelling in small object detection in the COCO-driving dataset, shows relatively poorer performance, with 1.3% lower mAPs than DINO. The proposed method achieves mAP and mAPs of 0.498 and 0.228, respectively, surpassing DINO by 1.4% and 3.6%, highlighting its superiority in detecting small objects and densely occluded objects. Compared with two-stage methods, the proposed method demonstrates a 15% and 20% higher Map than Faster-RCNN and Sparse-RCNN, respectively. Notably, for small object detection, the proposed method's mAP_*s*_ is 20% and 57% higher than Faster-RCNN and Saprse-RCNN, respectively. This underscores the advancement of the proposed method. Compared to single-stage methods, YOLOv7 achieves a 1.5% higher mAP_50_. However, the proposed method outperforms in mAP and mAP_*s*_ by 1.8% and 2.2%, respectively, surpassing YOLOv7 in detection accuracy. However, in parameter comparison, the method requires 1.87M more parameters and exhibits 2.7 times higher GFLOPs than YOLOv7, indicating higher storage and computational costs for the proposed method.

**Table 3:** Experimental results of the proposed method and other methods on the WiderPerdion dataset.

**Model**	**mAP_50_**	**mAP**	**mAP_*s*_**	**Epoch**	**Params**	**GFLOPs**	**FPS**
DAB-DETR (Liu S. et al., 2022)	0.671	0.447	0.164	50	43.87M	94	17
DN-DAB-DETR (Li F. et al., 2022)	0.699	0.414	0.132	50	43.47M	101	16
Deformable-DETR (Zhu et al., 2020)	0.749	0.465	0.191	50	39.85M	196	22
DN-Deformable-DETR	0.759	0.471	0.217	50	47.18M	265	23
DN-DAB-Deformable-DETR	0.761	0.472	0.204	50	47.21M	273	15
DINO (Zhang et al., 2022)	0.723	0.491	0.220	24	46.67M	279	23
Faster-RCNN (Ren et al., 2015)	0.716	0.431	0.189	36	41.32M	180	26
Sparse-RCNN (Sun et al., 2021)	0.693	0.412	0.145	36	41.35M	87	23
YOLOX-l (Ge et al., 2021b)	0.766	0.461	0.212	100	54.21M	155	69
YOLOv7 (Wang et al., 2023)	0.797	0.489	0.223	100	36.91M	103	161
Proposed method	0.785	0.498	0.228	24	38.78M	284	18

The experimental results of the proposed method on the existing advanced methods in the KITTI autonomous driving dataset are shown in Table 4. The KITTI dataset has been widely recognized in the field of autonomous driving, so the detection results on this dataset can prove the robustness and advancement of the method in this study to a certain extent. Compared with the Transformer-based object detection method, the detection accuracy of the proposed method is still the highest, and mAP_50_, mAP, and mAP_*s*_ are 2.3%, 3%, and 5.4% higher than the original method (DINO), respectively. In the two-stage object detection method, Faster-RCNN has the best detection performance, and the mAP_50_ and mA of the proposed method are 1.2% and 9.9 higher than that of Faster-RCNN, respectively. However, the mAP_*s*_ of the proposed method is 0.002 lower than that of Faster-RCNN. The object detection accuracy of the proposed method is still significantly ahead of Faster-RCNN. In the comparison of single-stage object detection methods, the mAP_50_ of YOLOv7 is still 2.6% higher than that of the proposed method, but the mAP and mAP_*s*_ of the proposed method are 0.5 and 3.9% higher than those of YOLOv7, which proves that the proposed method has better detection performance for small objects. Although the proposed method is ahead of YOLOv7 in terms of detection accuracy, the FPS of the proposed method is only 18, while the FPS of YOLOv7 is 103, which is far lower than the detection speed of YOLOv7. Therefore, how to further improve the detection speed of the proposed method is an important direction for future research.

**Table 4:** Experimental results of the proposed method and other methods on the KITTI dataset.

**Model**	**mAP_50_**	**mAP**	**mAP_*s*_**	**Epoch**	**Params**	**GFLOPs**	**FPS**
DAB-DETR (Liu S. et al., 2022)	0.731	0.421	0.172	50	43.87M	94	17
DN-DAB-DETR (Li F. et al., 2022)	0.752	0.432	0.197	50	43.47M	101	16
Deformable-DETR (Zhu et al., 2020)	0.778	0.472	0.212	50	39.85M	196	22
DN-Deformable-DETR	0.832	0.547	0.432	50	47.18M	265	23
DN-DAB-Deformable-DETR	0.850	0.551	0.431	50	47.21M	273	15
DINO (Zhang et al., 2022)	0.849	0.559	0.423	24	46.67M	279	23
Faster-RCNN (Ren et al., 2015)	0.858	0.524	0.448	36	41.32M	180	26
Sparse-RCNN (Sun et al., 2021)	0.819	0.519	0.418	36	41.35M	87	23
YOLOX-l (Ge et al., 2021b)	0.863	0.547	0.422	100	54.21M	155	69
YOLOv7 (Wang et al., 2023)	0.892	0.573	0.429	100	36.91M	103	161
Proposed method	0.869	0.576	0.446	24	38.78M	284	18

The experimental results comparing the proposed method with existing methods on the Waymo Open dataset are presented in Table 5. This dataset encompasses diverse traffic scenes, including nighttime driving and adverse weather conditions. Owing to the complexity and variability of the scenes, overall experimental results tend to be lower. Among Transformer-based object detection methods, DINO achieves the highest mAP and mAP50, while DN-DAB-Deformable-DETR records the highest mAPs. Compared to DINO, the proposed method exhibits an increase of 8.1%, 6.5%, and 27%, respectively in maps. Additionally, the proposed method's mAPs surpasses that of DN-DAB-Deformable-DETR by 2.1%. Additionally, the proposed method boasts lower parameter counts than those of the Transformer-based methods. However, due to the high computational complexity inherent in Transformers, the performance in terms of GFLOPs and detection speed (FPS) is suboptimal. The proposed method's detection speed is only a quarter of YOLOX's and significantly lags behind YOLOv7. Despite its higher detection accuracy compared to YOLOX and YOLOv7, the proposed method falls short of meeting practical requirements. Future research will focus on enhancing the detection speed of the proposed method.

**Table 5:** Experimental results of the proposed method and other methods on the Waymo open dataset.

**Model**	**mAP_50_**	**mAP**	**mAP_*s*_**	**Epoch**	**Params**	**GFLOPs**	**FPS**
DAB-DETR (Liu S. et al., 2022)	0.476	0.229	0.031	50	43.87M	94	17
DN-DAB-DETR (Li F. et al., 2022)	0.499	0.247	0.042	50	43.47M	101	16
Deformable-DETR (Zhu et al., 2020)	0.556	0.311	0.063	50	39.85M	196	22
DN-Deformable-DETR	0.522	0.387	0.092	50	47.18M	265	23
DN-DAB-Deformable-DETR	0.537	0.394	0.139	50	47.21M	273	15
DINO (Zhang et al., 2022)	0.623	0.398	0.111	24	46.67M	279	23
Faster-RCNN (Ren et al., 2015)	0.557	0.346	0.068	36	41.32M	180	26
Sparse-RCNN (Sun et al., 2021)	0.549	0.328	0.074	36	41.35M	87	23
YOLOX-l (Ge et al., 2021b)	0.653	0.414	0.115	100	54.21M	155	69
YOLOv7 (Wang et al., 2023)	0.675	0.421	0.124	100	36.91M	103	161
Proposed method	0.674	0.424	0.142	24	38.78M	284	18

#### 4.4.2 Visual analysis

**Figure 13:**
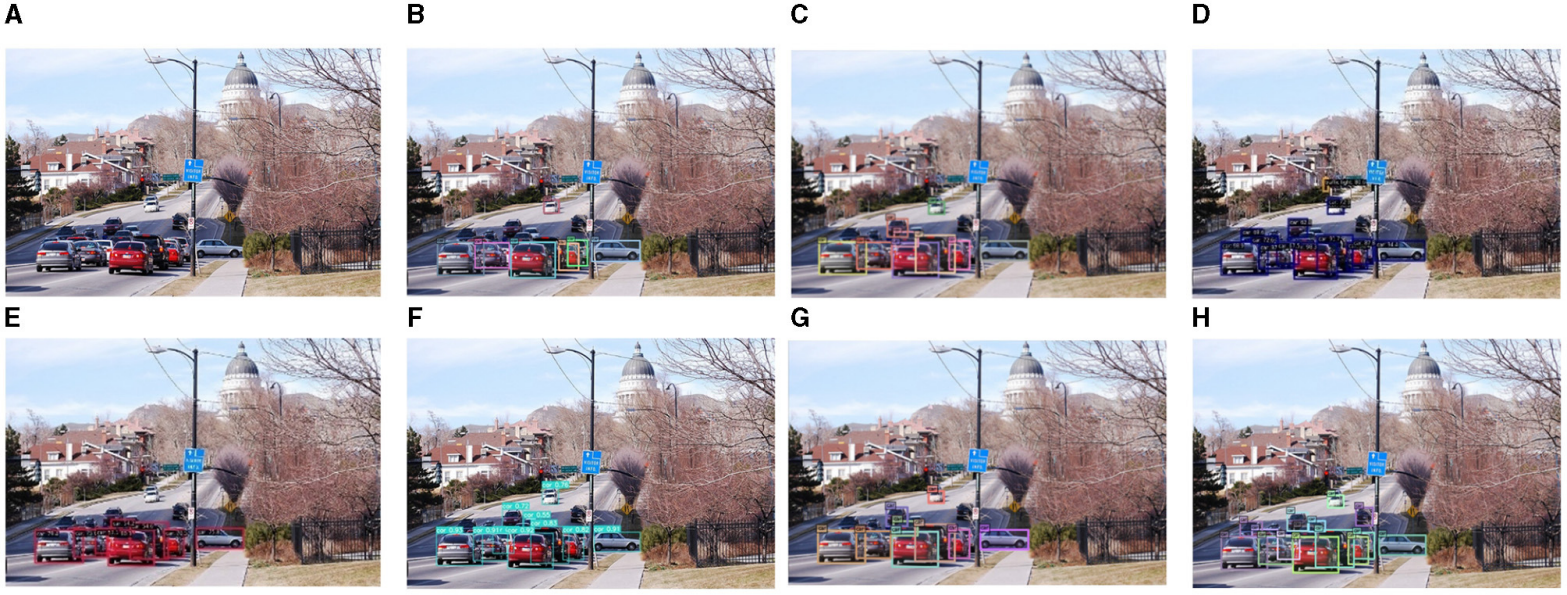
Comparison of the detection performance of the proposed method and other object detection methods on the COCO-driving dataset for small objects. **(A)** is the original image, **(B)** is the detection image of DN-DAB-DETR, **(C)** is the detection image of Deformable-DETR, **(D)** is the detection image of Sparse-RCNN, **(E)** is the detection image of YOLOX, **(F)** is the detection image of YOLOv7, **(G)** is the DINO model detection image, **(H)** is the detection image of the proposed method.

**Figure 14:**
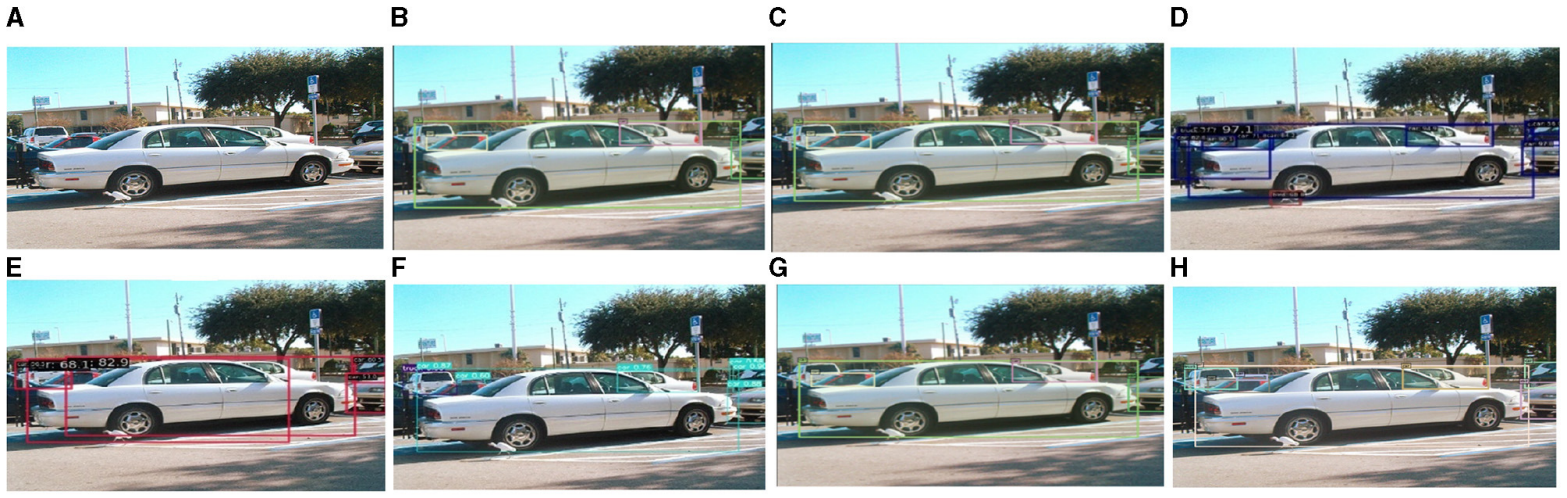
Comparison of the detection performance of the proposed method and other object detection methods on the COCO-driving dataset for dense occlusion objects. **(A)** is the original image, **(B)** is the detection image of DN-DAB-DETR, **(C)** is the detection image of Deformable-DETR, **(D)** is the detection image of Sparse-RCNN, **(E)** is the detection image of YOLOX, **(F)** is the detection image of YOLOv7, **(G)** is the DINO model detection image, **(H)** is the detection image of the proposed method.

**Figure 15:**
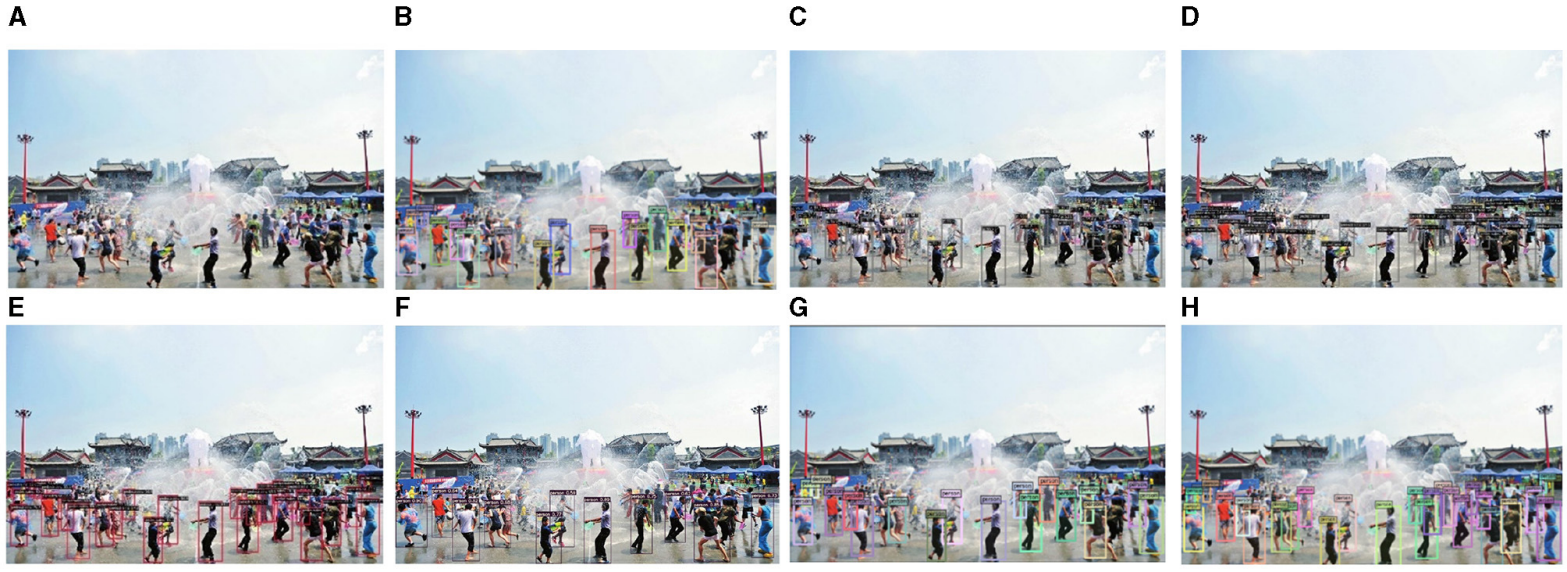
Comparison of the detection performance of the proposed method and other object detection methods on the WiderPerson dataset for small and dense occlusion objects is illustrated as follows: **(A)** denotes the original image, **(B)** represents the detection image of DN-DAB-DETR, **(C)** illustrates the detection image of Faster-RCNN, **(D)** represents the detection image of Sparse-RCNN, **(E)** denotes the detection image of YOLOX, **(F)** denotes the detection image of YOLOv7, **(G)** represents the DINO model detection image, and **(H)** represents the detection image of the proposed method.

**Figure 16:**
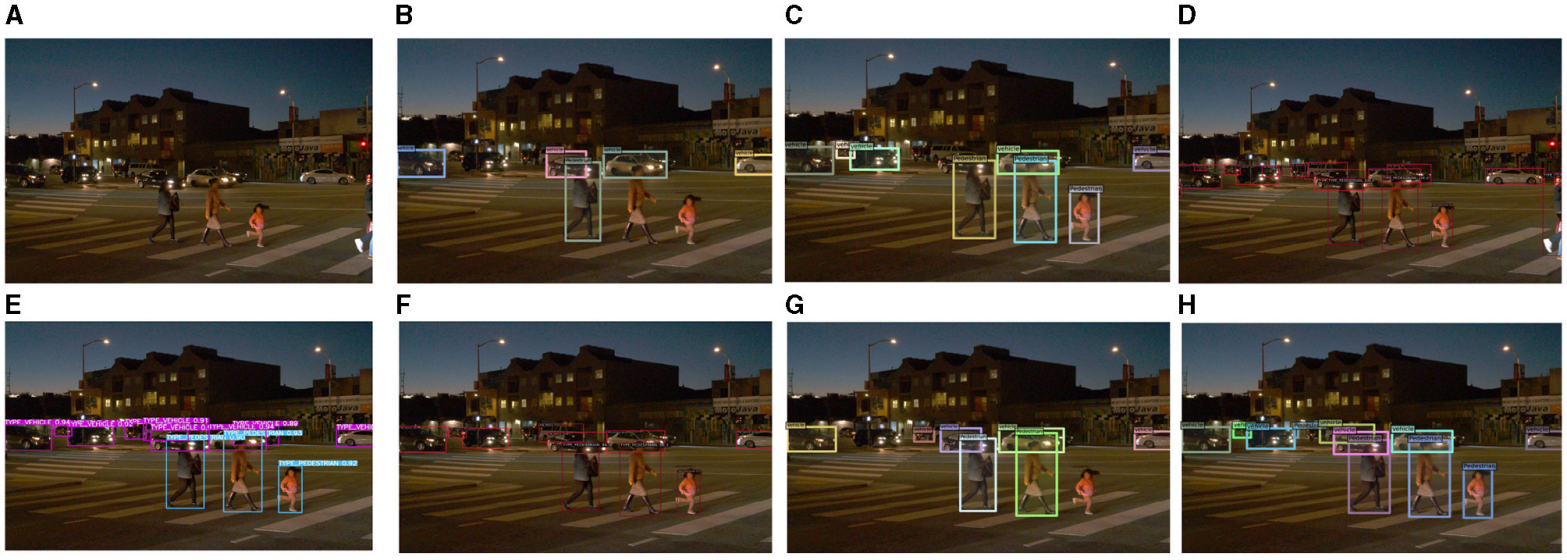
Comparison of the detection performance of the proposed method and other object detection methods on the Waymo Open dataset is illustrated as follows: **(A)** represents the original image, **(B)** denotes the detection image of DN-DAB-DETR, **(C)** illustrates the detection image of Deformable-DETR, **(D)** indicates the detection image of Sparse-RCNN, **(E)** represents the detection image of YOLOX, **(F)** denotes the detection image of YOLOv7, **(G)** indicates the DINO model detection image, and **(H)** represent the detection image of the proposed method.

**Figure 17:**
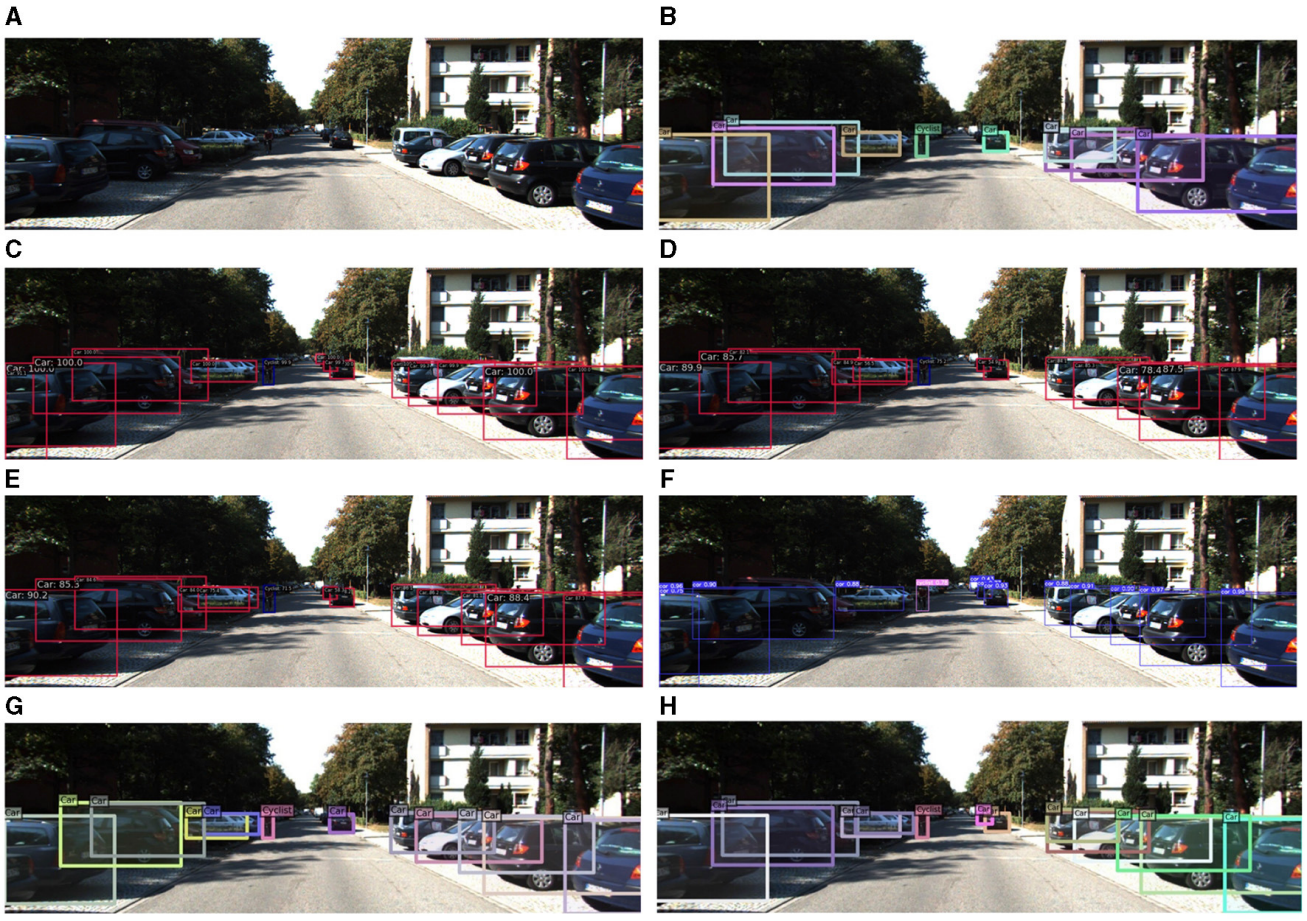
Comparison of the detection performance of the proposed method and other object detection methods on the KITTI dataset is illustrated as follows: **(A)** depicts the original image, while **(B)** illustrates the detection image of DN-DAB-DETR. Additionally, **(C)** represents the detection image of Faster-RCNN, **(D)** depicts the detection image of Sparse-RCNN, **(E)** shows the detection image of YOLOX, **(F)** illustrates the detection image of YOLOv7, **(G)** displays the DINO model detection image, and **(H)** showcases the detection image of the proposed method.

To further demonstrate the robustness of the proposed method in detecting unmanned driving scenarios, we compared it with existing methods and visualized the results, setting the confidence threshold to 0.5. The detection performances are depicted in Figures 13–17. Analysis of the results reveals that other methods often fail to detect objects when they are either too small or heavily occluded. Conversely, the proposed method effectively addresses the these challenges, demonstrating detection performance even for some small and densely occluded objects.

In Figure 13, the vehicle object appears small and heavily occluded, resulting in different degrees of false and missed detections. However, the proposed method effectively addresses these challenges by employing the query and noise reduction training method based on Gaussian attenuation, along with the matching method based on optimal transmission and Hungarian fusion. This approach approximately the occurrence of false positives and missed detection. For example, the black car under the right street light was only successfully detected by the proposed method. Specifically, DN-DAB-DETR, DN-Deformable-DETR, Sparse-RCNN, YOLOX, YOLOv7, and DINO detected seven, eight, 13, nine, 11, and 10 objects, respectively. However, the proposed method detected 14 objects, highlighting its superiority in detecting small objects and densely occluded objects.

As shown in Figure 14, owing to the large proportion of white vehicles, several vehicles are occluded, posing a significant challenge to object detection. For example, DN-DAB-DETR, Deformable-DETR, and DINO only detected four objects, YOLOX detected six, and the proposed method, Sparse-RCNN, and YOLOv7 detected seven. Considering that eight objects exist in the graph, the detection results of the proposed method are acceptable.

As depicted in Figure 15, the image contains numerous densely occluded objects, presenting significant challenges to the object detection process owing to the presence of various objects and mutual occlusions. For example, DN-DAB-DETR, Faster-RCNN, Sparse-RCNN, YOLOX, YOLOv7, detected 18, 23, 25, 22, and 12 objects, respectively. This indicates that the YOLOv7 method exhibits a relatively poor detection effect on densely occluded objects. Conversely, the method proposed in this study enhances the number of positive samples by using a hybrid matching method based on optimal transmission and Hungarian algorithm, thereby obtaining more positive sample information. Consequently, this approach effectively addresses the problem of false positives and missed detections of densely occluded objects. Finally, the method employed in this study achieves the detection of 27 objects, thereby attaining optimal detection results.

As illustrated in Figure 16, the image was captured at night, and the object features were not apparent owing to insufficient light. This resulted in significant challenges to the object detection task. For example, the girl at the zebra crossing, DN-DAB-DETR, and DINO were not successfully detected. Finally, DAB-DETR, Deformable-DETR, Sparse-RCNN, YOLOX, YOLOv7, DINO detected 5, 8, 12, 12, 10, and 7 objects, respectively. Additionally, the proposed method detected 11 objects. This finding proves that the proposed method still has superior robustness in complex traffic scenes.

As shown in Figure 17, considering that the left vehicle in the figure is in the shadow of the trees and the vehicles occlude each other, it is challenging to detect. Finally, DAB-DETR, Faster-RCNN, Sparse-RCNN, YOLOX, YOLOv7, and DINO detected nine, 14, 13, 12, 13, and 12 objects, respectively. The method in this study improves the feature extraction ability of the model by fusing the multi-scale Transformer feature extraction method using channel attention. Subsequently, it improves the detection accuracy and detects 14 objects, highlighting the advancement and robustness of the study's method.

The method employed in this study is tested oacross various datasets including KITTI, WiderPerson, and Waymo Open. The Waymo Open dataset, notable for its large volume and diverse unmanned driving scenes featuring and the method in this study always has the highest detection accuracy. This indicates that the proposed method has better robustness to some extent.

## 5 Discussion

Addressing the challenge of low detection accuracy for multi-scale changing, small, and densely occluded targets in complex traffic environments, this study proposes an improved object detection method utilizing Transformer and conducts experiments on COCO-driving, WiderPerson, KITTI, and Waymo open datasets. The experimental results consistently demonstrate that the superior target detection accuracy of the proposed method than existing advanced methods, revealing its robustness and advancement. However, practical implementation considerations, particularly considering the performance of unmanned driving perception system hardware, reveal the following limitations of the method:

1. Despite the high detection accuracy of the proposed method, its inference time is prolonged, resulting in subpar real-time performance owing to hardware limitations in the unmanned driving perception system.
2. The method exhibits high computational complexity, necessitating computational resources for both training and inference processes.
3. While the method in this study features a relatively small number of parameters, it is still slightly large, potentially consuming excessive storage space and memory. This could adversely affect the multi-task execution ability of the system.

Therefore, the focal point of future research will be on reducing the computational complexity of the model and improving the detection speed of the proposed method, while simultaneously ensuring the object detection's accuracy. For example, knowledge distillation can be used to reduce the number of parameters and improve the detection speed.

## 6 Conclusions

To address the problems existing in unmanned driving object detection methods, we proposed an improved object detection method for unmanned driving leveraging Transformers. First, a multi-scale Transformer feature extraction method, fused with channel attention, addresses suboptimal detection of multi-scale changing objects. Second, we employ a training method for query denoising using Gaussian decay to enhance small object detection accuracy. Third, a hybrid matching method combining optimal transport and Hungarian algorithms resolves missed and false detections of densely occluded objects. This study adopts a method based on the DINO algorithm. The experimental results demonstrate the effectiveness of the proposed method.

Experiments are conducted on COCO-driving, WiderPerson, KITTI, and Waymo datasets, comparing the proposed method with DINO-DETR, YOLOv7, and other object detection algorithms. The experimental results indicate that the proposed method achieves the highest object detection accuracy. However, analysis of GFLOPs and FPS reveals that while the proposed method significantly improves detection accuracy for multi-scale changing objects, small objects, and densely occluded objects, it also requires substantial computing resources, resulting in slow training and inadequate real-time performance. Therefore, future research will focus on reducing computational complexity, accelerating training speed, and improving real-time performance of the model.

## Data availability statement

The original contributions presented in the study are included in the article/supplementary material, further inquiries can be directed to the corresponding author.

## Author contributions

HZ: Writing – original draft, Writing – review & editing, Conceptualization, Data curation, Formal analysis, Funding acquisition, Investigation, Methodology, Project administration, Resources, Supervision, Validation. XP: Conceptualization, Data curation, Formal analysis, Methodology, Software, Visualization, Writing – original draft, Writing – review & editing, Investigation, Validation. SW: Data curation, Formal analysis, Investigation, Methodology, Software, Validation, Visualization, Writing – review & editing. J-BL: Conceptualization, Formal analysis, Funding acquisition, Project administration, Resources, Writing – review & editing, Supervision. J-SP: Conceptualization, Funding acquisition, Project administration, Resources, Supervision, Writing – review & editing. XS: Data curation, Funding acquisition, Resources, Supervision, Writing – review & editing. XL: Conceptualization, Data curation, Formal analysis, Funding acquisition, Investigation, Methodology, Project administration, Resources, Supervision, Writing – original draft, Writing – review & editing.
